# Family-Reported Outcomes Measures (FROMs) Screening Programs an Integral Part of Chronic Disease Management: A Scoping Review

**DOI:** 10.1177/10732748251339217

**Published:** 2025-06-19

**Authors:** Sylvie Lambert, Li-Anne Audet, Lydia Ould Brahim, Jamie Schaffler, Cecilia Garcia Ramirez, Francesca Frati, Sonya Sangha, A. Fuchsia Howard, Leah Lambert

**Affiliations:** 1 Ingram School of Nursing, McGill University, Montreal, QC, Canada; 2 St. Mary’s Research Centre, Montreal, QC, Canada; 3 Schulich Library of Physical Sciences, Life Sciences, and Engineering, McGill University Libraries, McGill University, Montreal, QC, Canada; 4 BC Cancer Provincial Health Services Authority, Vancouver, BC, Canada; 5 School of Nursing, University of British Columbia, Vancouver, BC, Canada

**Keywords:** caregivers, unmet needs, family-reported outcomes (FROs), family-reported outcome measures (FROMs), scoping review, screening programs, chronic disease, cancer, patient-reported outcomes (PROs), distress screening

## Abstract

Caregivers experience physical, psychological, emotional, and practical challenges resulting from their roles and responsibilities. These challenges negatively impact caregivers’ health and well-being. Screening for patient-reported outcomes (PROs) is now considered a cornerstone of chronic disease management to improve symptom identification and management, quality of life, and survival. Similarly, screening for family-reported outcomes (FROs) could help promote caregivers’ health and well-being. Though family-reported outcome measure (FROM) screening programs have emerged, the nature and extent of their development and evaluation in the context of chronic disease remains unknown. This scoping review aimed to identify the extent to which FROM screening programs among caregivers of adults with a chronic disease have been developed and evaluated. PRISMA-ScR and the methods recommended by the Joanna Briggs Institute were followed. Four electronic databases (Ovid- Medline(R), Ovid- Classic + Embase, Ovid-APA PsycInfo 1967-onwards, CINAHL Plus with Full Text, and ProQuest Dissertations and Theses) were searched iteratively to identify published literature describing FROM screening programs. Secondary search strategies and a search of grey literature were also undertaken. Data were extracted using a standardized table and analyzed using descriptive statistics and qualitative content analysis. A total of 38 studies describing 17 unique FROM programs were evaluated. Studies were published between 1999-2024 and primarily from Australia (n = 11), the United States (n = 8), and the United Kingdom (n = 6). Caregivers included (n = 4312) were most commonly spouses of patients with cancer. Screening was primarily used to tailor interventions (rather than monitor symptoms) and focused on caregivers’ needs (e.g., information, managing patient symptoms). Nurses typically responded to the screening. Most programs offered three types of follow-up: informational/educational resources, referrals to specialists or community groups, and/or real-time discussion and feedback with the interventionist. Although the FROM programs positively impacted proximal variables (e.g., preparedness), this did not translate to more distal outcomes (e.g., quality of life, anxiety). Future research on the timing of screening, caregiver engagement, and efficacious follow-up interventions is needed.

## Background

Now responsible for 75% of deaths worldwide, chronic diseases are the leading cause of disease burden globally.^1^ As chronic disease incidence continues to rise, care is increasingly provided by unpaid caregivers, often family members or friends, who, on average, are older than in previous decades.^2,3^ These caregivers usually take on multiple care roles, including helping care recipients with their activities of daily living.^4^ Due to their caregiving roles, caregivers may experience adverse negative outcomes such as high levels of physical, psychological, practical, or social burden, ultimately decreasing their physical and mental health and well-being^5^ and ability to continue their vital caregiving role.

Patient-Reported Outcomes (PROs) are defined as any account of a patient’s health status (e.g., symptoms experienced) that is provided directly by the patient (also referred to as care recipient), without it being interpreted by a clinician.^6^ PROs are measured using standardized tools referred to as patient-reported outcome measures (PROMs). Screening patients’ health status using PROMs has been shown to have a wide range of benefits for patients (e.g., improved health-related quality of life (QoL), decreased symptom severity)^7^ and clinicians (e.g., saving time during consultations).^8,9^

Along the same lines, Family-Reported Outcomes (FROs) can be defined as consequences of caregiving on the lives of caregivers, such as their QoL or level of distress, as declared by caregivers.^10^ With the growing recognition of caregivers’ needs and the challenges arising from their role, increased attention is being given to screening for caregivers’ health status using Family-Reported Outcomes Measures (FROMs) and their implementation in routine care.^11-13^ However, FROM screening programs have not been as extensively and consistently studied and implemented as PROM programs, and FROM screening is far from being the standard of care.^14^ As a result, FROM screening still presents some challenges, such as the availability of valid and reliable screening measures^15^ and lack of clarity regarding who should be responsible for following up on the results of FROM screening. Additionally, the specific components of FROM screening programs and their impact on caregivers are largely unknown.

Due to these knowledge gaps, this scoping review aimed to identify the extent to which FROM screening programs among caregivers of adults with a chronic disease have been used. This includes identifying key components of FROM screening programs (henceforth FROM programs), namely (a) the follow-up care offered after FROM screening, (b) the type of FROs captured and the measures used, (c) the characteristics of the caregivers targeted by FROM programs, (d) the barriers and facilitators to FROM program implementation, and (e) the effects of these programs on caregivers’ health outcomes.

## Methods

A scoping review was chosen to map the literature on FROM programs and identify key knowledge gaps.^16^ Methods adhered to guidance from the Joanna Briggs Institute and the Preferred Reporting Items for Systematic Reviews and Meta-Analyses—Scoping Review Extension (PRISMA-ScR).^17,18^ The protocol was registered on September 18^th^, 2021 (https://osf.io/daqnm).

### Inclusion and Exclusion Criteria

#### Participants and context

Caregivers were defined as those providing unpaid care and support, such as physical care, administrative tasks, and/or emotional support to someone living with a chronic disease.^19^ Considering the different environments and contexts in which caregiving can occur, no restrictions were placed on the type of care or support provided by the caregiver, or the number of hours spent caregiving. Similarly, the search criteria included all types of settings, such as care recipients’ homes, community clinics, and hospitals. Key exclusion criteria were if the caregiver was paid, under 18 years of age, or caring for someone under 18 years of age. Chronic disease was broadly defined as a health condition that is not communicable, can usually be managed but not cured,^20^ can be mental and/or physical, and tends to be of long duration.^1^ Conditions excluded were injuries following an accident, both physical and intellectual developmental issues, and any communicable diseases.

#### Concept

*FROM programs* were defined as any planned attempt to purposefully collect FROMs among eligible participants and where the results of the screening would be reviewed by a clinician (or dedicated person) with the goal or expectation of putting in place interventions or follow-up care to respond to the results of the screening. Programs were included if caregivers self-completed at least one FROM; a checklist or other measure developed to be completed by a clinician was not eligible. FROMs could be across any domain of caregivers’ health, functioning, and/or quality of life. No restrictions were placed on the type of clinician responding to the FROM screening.

Programs focusing on PROMs were excluded. Studies in which FROMs were used to examine the prevalence of health issues or the inter-relationships among these for descriptive purposes were excluded, as were studies developing or adapting FROMs only (rather than a FROM program). Psychometric evaluations of FROMs and studies focusing on outcomes related to clinicians were also excluded.

#### Type of Studies

In keeping with the goal of a scoping review, all study designs were included. Conference proceedings and literature syntheses were excluded. Protocols were excluded unless a findings paper was published, and the protocol served to ensure all relevant data were extracted. French and English studies were included. No limit on time since publication was applied.

### Search Strategy and Study Selection

The search was conducted using a three-step iterative process in Ovid—Medline(R), Ovid—Classic + Embase, Ovid-APA PsycInfo 1967-onwards, CINAHL Plus with Full Text, and ProQuest Dissertations and Theses Global.^17,18^ The search began in July 2021 and was then updated in June 2023.

#### Step 1

The initial Medline search strategy was developed by a medical librarian (FF) and two authors (SL, CGR). The search comprised a combination of subject headings and keywords based on the two main concepts: FROMs and chronic disease. The Medline search was peer-reviewed by a second medical librarian using the PRESS guidelines^21^ and the feedback was incorporated prior to running the search. Duplicates were removed based on the guidelines published by Bramer et al.^22^ Then, records were imported into the Rayyan QCRI web application and remaining duplicates were removed as titles were screened. A training session with a random sample of 35 titles took place between two authors (LAA and SL). The training involved reviewing each title and discussing their inclusion/exclusion. After this training, each author independently screened 200 titles. As the Gwets AC coefficient was 0.94 (95% CI: 0.91-0.98), the authors independently screened the remaining titles.

Following this, the abstracts of included titles were screened by one author (LAA). To ensure rigor, a second author (SL) reviewed abstracts retained from the Ovid- Medline(R) search. Reasons to exclude abstracts were labelled. Full texts were screened for inclusion by one author (LAA) and all included full texts were verified by a second author (SL). Twenty-one full texts were retained (2 of which were subsequently rejected). Throughout this initial search, the inclusion/exclusion criteria were refined, particularly pertaining to the definition of FROM programs.

#### Step 2

The librarian (FF) analyzed the full texts retrieved from Step 1 using the Yale MeSH analyzer^23^ and revised the initial search strategy. The full search strategy, including the initial and updated search datasets, was published (https://doi.org/10.5683/SP3/ZZIRT0).

#### Step 3

No changes were made to the search following Step 2. The librarian (FF) then translated the revised search into remaining databases, including rerunning the search in Medline. Duplicates were removed as per Step 1 with the additional removal of records already screened. As part of the grey literature search, Proquest Dissertations and Theses database was searched. The overall study selection followed the process described in Step 1. Inter-rater reliability was established among the four authors who screened titles (SL, LAA, LOB, CGR) with a random sample of 200 titles and then inter-rater reliability was re-established at the abstract stage with 50 abstracts. The Gwet’s AC coefficients ranged from 0.85 (95% CI, 0.73-0.98) to 0.91 (95% CI, 0.87-0.94). If there was uncertainty, the title or abstract was retained to be reviewed further at the next screening stage.

### Data Extraction

The data extraction table included the characteristics of the retained studies (e.g., authors, country, sample size) and specific information about each FROM program (e.g., FROMs and category of FROMs, interventions, efficacy measures). The table was revised and adjusted throughout the extraction process. Data were extracted by one author and verified by at least one other author. When changes were made to the data extraction table, the previously extracted studies were re-read for the additional data to be extracted. When a required piece of information was missing, the information was requested directly from study authors by e-mail. If there was no response, the missing information was coded as “not specified.”

### Data Analysis

All data were summarized and descriptive statistics calculated using Microsoft Excel. For the impact of FROM programs, the results of only randomized controlled tirals (RCTs) and quasi-experimental studies were summarized. If outcomes were reported by feasibility/acceptability and pilot studies these were excluded from the findings as is not the purpose of these types of studies. All outcomes reported by at least three studies were included in the results section and grouped according to short- (≤1 month), medium- (>1 month to >6 months), or long- (>6-months) term. Study designs were grouped into overarching categories: RCTs, quasi experimental, pre-experimental, acceptability and feasibility, process analysis, and observational cohort studies. Qualitative data were summarized using descriptive inductive content analysis.^24^ Data were open coded (brief descriptive label applied to relevant data) and grouped into initial sub-categories. Following this, sub-categories were reviewed and refined and further grouped into broader overarching categories. Uncertainties were discussed with other team members and refinements to the categories made as team members commented on drafts of the findings.

## Results

**Figure 1.**
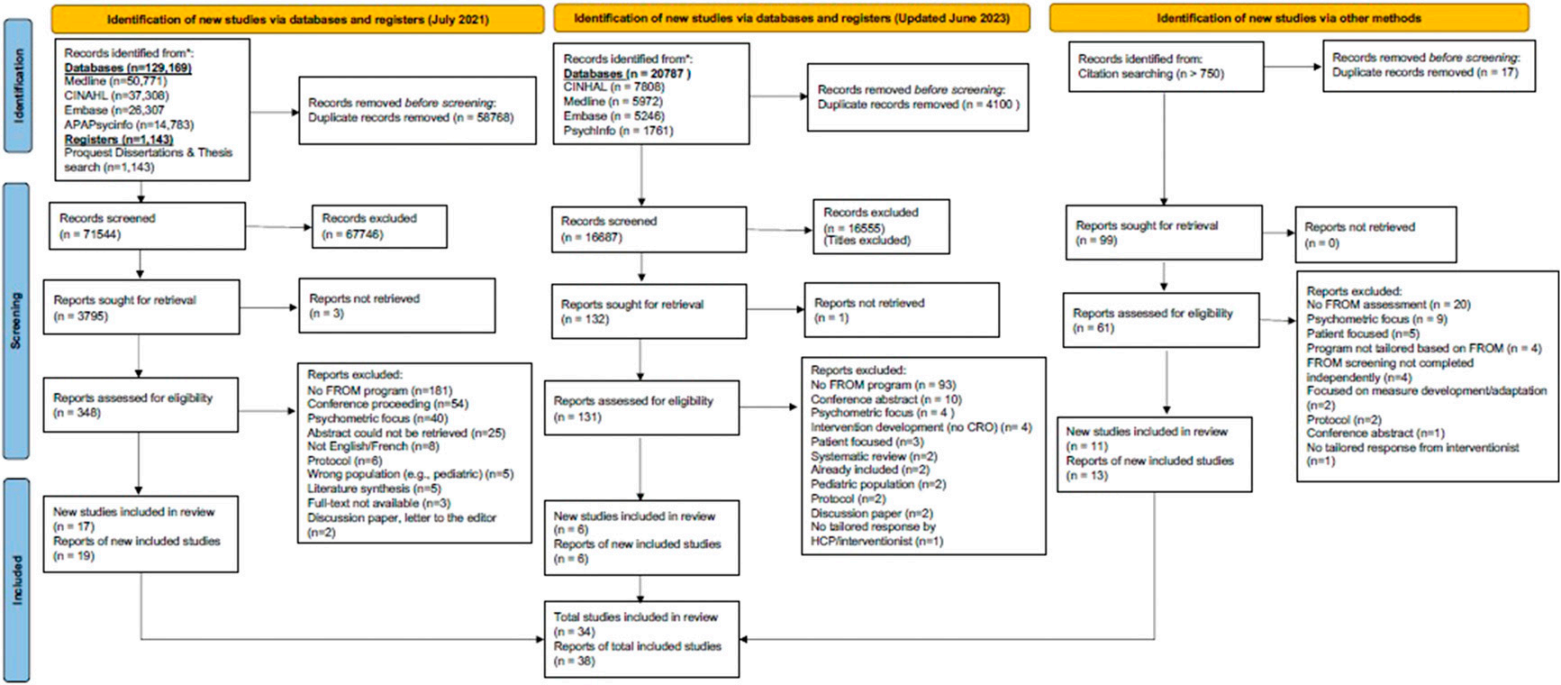
PRISMA Flow Diagram.

Of the 88 231 titles screened, 3927 abstracts were screened, and 375 were included in the full-text review. From these, 25 studies were retained in addition to 13 studies identified from the secondary search strategy. Across the 38 studies reviewed,^12,25-60^ 17 distinct FROM programs were described (many studies used the same programs in different settings). Further details are provided in the PRISMA Figure 1.

### Overview of Studies

#### Types of Studies

Table 1 summarizes the included studies, which were mostly from Australia (n = 11),^27,29,30,41,43,45,48,49,53,58,59^ the United States (n = 8),^31,34,38,44,54-56,60^ or the United Kingdom (n = 6).^33,36,37,39,40,52^

**Table 1.** Overview of Studies.

Author, Year, Country	Study design	Aim(s)	Setting & sample	Overview of from program	Key findings
**1. Aoun et al., 2015a**^29^ **Australia** (Aoun et al, 2015a,b,c^25,26,28^ and Aoun 2018^30^ a related reports)	Quasi-experimental (stepped-wedge cluster non-randomized trial)	To examine the impact on family CG outcomes of using the carer support needs assessment tool (CSNAT) intervention to identify and address support needs in end-of-life home care	**Setting:** Three sites of the largest provider of home-based palliative care **Population**: Primary adult family CGs of terminally ill adults (with cancer or non-cancer diagnoses) **Sample**: N = 620 CGs (T = 441; C = 179) **Mean age** (years): 63.0 ± 12.9 **% female**: 73.0% **Race/ethnicity:** 58.7% Australian **Caregiver relationship:** 68.3% spouse/partner **Patient illness:** Cancer (74.8%); cancer + non-cancer diagnosis (14.6%); non-cancer diagnosis (10.6%) **Duration of caregiving:** 24.7 months ± 39.3 **Mean # of hours/week caregiving:** Not specified	**Program description:** At least two nurse visits using the CSNAT practitioner-facilitated but CG-led approach to needs assessment, which included (1) introduction of the tool and assessment of the CGs’ needs using the CSNAT, which covers 14 support domains; (2) a conversation between the CG and the practitioner (here nurse) aimed at identifying CG’s individual support needs and priorities; and (3) collaborative development of a shared action plan tailored to the CG’s needs and, when possible, shared review of the plan (^a^see note at bottom of table for regarding CSNAT update). **Control:** Usual care included staff meetings with the CG during the client visit and discussing CG needs on an informal basis. The staff member then offer services and equipment that Silver Chain was able to provide. **Duration:** Not specified **Intensity:** At least 2 nurse visits with CGs, 2-3 weeks apart **Frequency of FROM screening:** At least twice (baseline and 2-3 weeks later) **Clinician responding to FROMs:** Nurses **Alert system**: Not specified	**Primary:** T > C for reduction in CG strain T = C for CG distress **Secondary:** T = C for CG mental and physical wellbeing and workload
**2. Aoun et al, 2015b**^25^ **Australia** (Aoun et al, 2015a,b,c^25,26,28^ and Aoun 2018^30^ a related reports)	Acceptability and feasibility study	To describe family CGs of terminally ill adults' experience of the CSNAT intervention in the context of home-based palliative care	**Setting & Population**: Same as Aoun et al (2015a) **Sample:** N = 233 CGs (subset of Aoun et al 2015a) **Mean age** (years): 62.1 ± 12.4 **% female:** 70.4% **Race/ethnicity:** 55.4% Australian **Caregiver relationship**: 67.4% Spouse/partner **Patient illness:** Cancer (75.1%); cancer + non-cancer diagnosis (16.7%); non-cancer diagnosis (8.2%) **Duration of caregiving**: Median = 10 (0.1-420) **Mean# of hours/week caregiving**: Not specified	Same as Aoun et al. (2015a)^29^	The majority of CGs reported the CSNAT assessment process were straightforward and easy. Four key themes were identified: **1) Practicality/usefulness of CSNAT:** CSNAT raised issues that may not have otherwise identified and provided a format to express support needs. **2) Emotional responses:** Evoked a spectrum of emotional responses (e.g. confrontation about CG situation, humbling, considering future needs). **3) Validation, reassurance & empowerment**: Process was almost universally welcomed because of support provided in response but also because it validated CG role. **4) Accessing support and how this was experienced**: Support strategies implemented helped to meet family CGs’ informational and emotional needs.
**3. Aoun et al, 2015c**^26^ **Australia** (Aoun et al, 2015a,b,c^25,26,28^ and Aoun 2018^30^ a related reports)	Acceptability and feasibility study	To describe nurses' feedback on and experience of the implemention of the CSNAT intervention with family CGs in home-based palliative care	**Setting:** Same as Aoun et al (2015a) **Population**: Nurses who administered the CSNAT in Aoun et al (2015a) **Sample:** n = 44 nurses* (only CG socio-demographics reported in Table 1)	Same as Aoun et al. (2015a)^29^	Nurse feedback was positive in terms of perceived benefits in comparison to usual care. Using the CSNAT was described as providing guidance, focus and structure to facilitate discussion with CGs and as identifying needs and service responses that would not otherwise have been undertaken in a timely manner. 82% found CSNAT was effective in eliciting CGs concerns and helped them assess CG needs. 65% agreed that there were positives for them when using the CSNAT compared to standard practice. Nurses agreed that the actions taken were within the service capacity, if they related to assisting the CGs provide care (77%) compared to providing direct personal support for the CG (56%).
**4. Aoun et al, 2016** ^27^ **Australia**	Acceptability and feasibility study	To assess the feasibility and relevance of the CSNAT based on the perspectives of CGs of adults with motor neurone disease and their care advisors in the context of home-based care	**Setting:** Motor neurone disease Association of Western Australia **Population**: Adult primary family CGs of clients of the motor neurone disease Association of Western Australia and their care advisors **Sample:** N = 24 CGs **Mean age** (years): 63.8 ± 12.9 **% female:** 75% **Race/ethnicity:** 62.5% Australian; 29.2% British; 8.3% other (cultural background) **Caregiver relationship**: 79.2% Spouse/partner **Patient illness:** Motor neurone disease **Duration of caregiving**: Not specified **Mean # of hours/week caregiving**: Not specified	**Program description:** See Aoun et al (2015a)^29^ for description of CSNAT approach. Here undertaken with a care advisor (experience in nursing or physical therapy and works for the association provides home visits, case coordination, provides information and disability aids) **Control**: N/A **Duration:** 2 visits 6-8 weeks apart **Intensity:** 2 visits of unknown length; took median of 10 minutes (range 3-20) to complete the CSNAT form **Frequency of FROM screening:** Twice (during two visits) **Clinician responding to FROMs:** Care advisors (experience in nursing or physical therapy for 20 to 35 years) **Alert system**: Not specified	CSNAT approach for identifying and addressing CGs’ support needs was found relevant and acceptable by family CGs and care advisors. **CGs:** A CG-led assessment process gave them a sense of validation, reassurance, and empowerment, as reflected in their narratives. It allowed them to identify the highest support priorities as “knowing what to expect in the future,” “knowing who to contact if concerned,” and “equipment to help care” **Care advisor:** Found the approach more comprehensive and formalized compared to usual care. It allowed them to more clearly assess needs, offer a more structured follow-up, and to focus on the CG and family.
**5. Aoun et al., 2018a** ^30^ **Australia**	Acceptability and feasibility study	To identify and respond to the support needs of CGs of adults with dementia and, from their perspectives, assess the acceptability and relevance of using the CSNAT in the context of home-based care	**Setting:** Community care organisation **Population**: Family CGs of people living with dementia **Sample:** N = 21 CGs **Mean age** (years): 61 (Median = 60, 22-88) **% female:** 66.7% **Race/ethnicity**: 66.7% “Australian” **Caregiver relationship**: 42.9% Spouse/partner **Patient illness:** Dementia **Duration of caregiving**: Mean 49.0 months (range: 6-240) **Mean # of hours/week caregiving**: Not specified	**Program description:** See Aoun et al (2015a)^29^ for description of CSNAT approach. Here undertaken during two home visits with a client care advisor (provide support and coordinate services to adjust care and funding). **Control**: N/A **Duration:** 2 visits mean of 7.05 weeks apart (range 3-14 weeks) **Intensity:** 2 visits of unknown length (face-to-face visit and in some cases the second contact was completed by telephone) **Frequency of FROM screening:** Twice (once at each visit) **Clinician responding to FROMs:** Care advisors from the community care organization **Alert system**: Not specified	CGs provided positive feedback. Most CGs considered the CSNAT approach to be user-friendly. The CSNAT approach provided an opportunity for CGs to reflect on their situation. CGs appreciated that the CG-led approach was an opportunity to be listened to. Use of the CSNAT prompted CGs to articulate their needs for support, providing direction to the client care advisors to offer appropriate strategies.
**6. Aoun et al., 2018b**^28^ **Australia** (Aoun et al, 2015a,b,c^25,26,28^ and Aoun 2018^30^ a related reports)	Quasi experimental	To examine the extent to which use of the CSNAT intervention while caregiving affected bereaved CGs’ perceptions of their grief, well-being, adequacy of support received, and fulfilling their preferred place of death	**Setting:** Same as Aoun et al (2015a) **Population**: Same as Aoun et al (2015a) **Sample**: N = 212 CGs **Mean age** (years): 64.6 ± 11.18 **% female**: 75.5% **Race/ethnicity:** 61.8% Australian **Caregiver relationship:** 68.4% spouse/partner **Patient illness:** Cancer (71.7%); cancer + non-cancer diagnosis (16.0%); non-cancer diagnosis (12.3%) **Duration of caregiving:** Not specified **Mean # of hours/week caregiving:** Not specified	Same as Aoun et al. (2015a)^29^	**T3 (4-6 months post-bereavement):** T > C for perceived adequacy of meeting pre-bereavement support needs provided for CG **Secondary:** **T3 (4-6 months post-bereavement):** T > C for achievement of patients’ or CGs’ preferred place of death (e.g. hospice, home, hospital) and agreement on the preferred place of death between patients/CG **T3 (4-6 months post-bereavement):** T = C for mental and physical well-being and level of grief in bereavement
**7. Applebaum et al, 2024** ^31^ **United States**	2-arm RCT	To assess the feasibility, acceptability, and preliminary efficacy of pairing the CancerSupportSource^TM^-Caregiver (CSS-CG) intervention with a follow-up consultation in improving CGs’ unmet needs, axiety, depression, and quality of life (QoL)	**Setting:** One outpatient center; two inpatient floors of the main hospital and the Bone Marrow Transplant (BMT) clinic. **Population**: Adult family CGs (≥18 years old) to patients of Memorial Sloan Kettering cancer center (MSK) **Sample**: N = 150 CGs (T = 75; C = 75) **Mean age** (years): 52.3 ± 14.0 **% female**: 66.0% **Race/ethnicity:** 84% Caucasian **Caregiver relationship:** 70% spouse/partner **Patient illness:** Cancer **Duration of caregiving:** Most CGs were within their first year of caregiving **Mean # of hours/week caregiving:** 71% spent less than 8 hours/day on caregiving	**Program description:** CSS-CG screening plus consultation intervention consisting of an electronic distress screening and automatic referral system designed to address CGs’ concerns including their own self-care needs, emotional well-being, and caregiving tasks and concerns about the patients’ well-being. CGs could request pertinent educational materials for lower concern items; and could request to speak with someone about the need to receive a referral and/or receive information for higher concern items. An overall CG distress score and a depression risk subscale score (score ≥5 indicating risk for clinically significant depression) were calculated. Support included: (1) written report on item-level feedback including CGs' greatest concerns and their depression risk status; (2) tailored information and/or referrals based on CGs’ indicated need and depression score; and (3) individual consultation with a nurse or clinical research coordinator to review the report to assess change in distress and modify (add/remove/change) referrals as appropriate, and identify and problem solve barriers to accessing referrals. **Control:** Enhanced usual care which consisted of access to the MyMSK portal CG resources page, including links to MSK CG educational materials, contact information for psychosocial services, and external resources. **Duration:** Consultation call planned for 3-6 days post screening **Intensity:** Not specified **Frequency of FROM screening:** Once **Clinician responding to FROMs:** Nurses and clinical research coordinator **Alert system**: Participants who met criteria for being at risk for depression were evaluated within by a licensed clinical psychologist for evaluation and triage	T = C for unmet needs from baseline to 6-months T > C for QoL emotional well-being from baseline to 6-months T = C for global distress; QoL (total, physical, social/family, and functional well-being subscales); and anxiety and depressive symptomatology. Total score on the CSS-CG = 28.5 ± 19.9 (e.g. not at all and slight levels of concern) 38% of CGs met the risk threshold for clinically significant depression Top three concerns were: the patient’s pain/physical discomfort (61%); the patient’s cancer progressing or coming back (60%); and feeling nervous or afraid (43%). **Feasibility:** CSS-CG screening plus consultation intervention was feasible: Among 75 CGs randomized to treatment group, 66 (88%) completed CSS-CG and consultation. **Acceptability:** CSS-CG screening plus consultation intervention was helpful in connecting CGs to support and helping them feel cared for and integrated into cancer care.
**8. Aubin et al, 2021** ^32^ **Canada**	2-arm RCT	To assess the feasibility and preliminary effects of an intervention including systematic distress and problem screening as well as privileged contact with an oncology nurse in improving distress, QoL, caregivning preparedness, and perceived burden for CGs of adults with lung cancer	**Setting:** One cancer center **Population**: Family CGs of patients with nonsurgical lung cancer **Sample:** N = 109 CGs (T = 54; C = 55) **Mean age** (years): 61.8 ± 11.7 **% female:** 72.5% **Race/ethnicity:** 100% French speaking Caucasians **Caregiver relationship**: 68.9% Spouse/partner **Patient illness:** Lung cancer **Duration of caregiving**: Not specified. **Mean # of hours/week caregiving**: Not specified.	**Program description:** Family CGs underwent an initial assessment using a distress screening tool measuring distress, problems (e.g., practical, emotional), and symptoms at baseline. CGs then received 1 × 20-minute session with an oncology nurse to review the results and plan tailored problem-solving strategies. Session content included acknowledgement and discussion of CG’s emotions, counseling on the importance of self-care, and provision of community resources and educational materials related to the CG roles. Sessions focused on aiding the CG to become proactive and empowered members of the care team. For CGs reporting high distress, the oncology nurse liaised with the family physicians for additional help providing emotional support. **Control**: Usual care **Duration:** 9 months or until relative's death **Intensity:** Variable. All CGs completed minimum one distress and problem screening and had 1 × 20-minute session with nurse. 83% completed remining screenings, some had further appointments with the nurse or received higher intensity support **Frequency of FROM screening: Variable.** Every 2 months for the study duration **Clinician responding to FROMs:** Oncology nurses **Alert system**: Not specified	**Primary:** **T4 (9 months):** T = C on distress, anxiety and depression. **Secondary:** **T4 (9 months):** T > C on CG preparedness. T = C on psychological burden and QoL **Qualitative interviews:** 1) CGs found the intervention reassuring and they valued occasion to have privileged contact with an oncology nurse despite low consultation frequency with nurse (attributed to prioritizing patients’ well-being). 2) They appreciated expressing in writing emotions they often did not verbalize. They suggested using the distress screening tool at transition points (i.e., diagnosis, progression, and new symptom outbreak) rather than systematically every 2 months. 3) Mixed views on recommending the transfer of distress screening tool results to their family physician.
**9. Barrowclough et al, 1999** ^33^ **United Kingdom**	2-arm RCT	To evaluate the effectiveness of a needs-based family psychosocial intervention service for out-patients with schizophrenia and their CGs	**Setting:** Outpatient previously admitted to the mental health unit of two hospital trusts **Population**: CG of outpatients (aged 18-65) diagnosed with schizophrenia for^3^ 2 years who had been hospitalized for psychotic symptoms in the previous 2 years. **Sample**: N = 77 CGs (T = 39; C = 38) **Mean age** (years): 53 ± 14.28 **% female**: 71.4% **Race/ethnicity:** Not specified **Caregiver relationship:** Not specified; 22% of patients lived with a spouse/partner (not confirmed these were participating CGs) **Patient illness:** Schizophrenia **Duration of caregiving:** Not specified **Mean # of hours/week caregiving:** 44.4 ± 27.03 face-to-face contact between the patient and CG/week	**Program description:** Family support worker provided support in 5 domains: Information, benefits advice, advocacy, as well as emotional and practical support + tailored dyadic psychosocial interventions with a clinical psychologist, with collaboration of family support worker when possible, consisting of problem-solving techniques, cognitive-behavioural interventions with families, and/or individual cognitive- behavioural interventions with patients with psychosis. Aim was for patients to attend at least half of the sessions. **Control:** Family support intervention alone **Duration:** 6 months **Intensity:** Variable **(**frequency and content matched to CG responses). Minimum dose 10 sessions with psychologist**,** but 10 CGs did not achieve this. Of CGs who did, median = 14 sessions, range 10-19 + phone contact with family support worker (median = 4, range 0-15) + home visits with family support worker (median = 3, range 0-12) **Frequency of FROM screening:** Once **(**baseline) **Clinician responding to FROMs:** Clinical psychologist with collaboration of patient’ family support worker (provides information, advocacy, support) **Alert system**: Not specified	**Primary:** Patient outcomes: **T1 (6 months):** T = C relapses T > C for days spent in relapse CG outcomes: **T1 (6 months):** T > C reduction in the number cardinal needs and the number of problems **Secondary:** Patient outcomes: **T1 (6 months):** T > C global functioning T = C severity of schizophrenia symptoms, social functioning CG outcomes: **T1 (6 months):** T = C for distress and burden
**10. Bitz et al. 2014** ^34^ **United States**	Acceptability and feasibility study	To describe a gender strengths-based intervention, assess its feasibility in early treatment, and report on satisfaction and distress data for adults with breast cancer and their CGs	**Setting:** Medical center **Population**: Adults with breast cancer requiring surgical consultation and their primary CG (preferably spouse/partner) **CG Sample:** N = 82 **Mean age** (years): Not specified **% female:** 23% **Race/ethnicity:** Not specified **Caregiver relationship**: 68% of patients were married or had a life partner (likely these were the CG participants) **Patient illness:** Breast cancer **Duration of caregiving**: Not specified **Mean # of hours/week caregiving**: Not specified	**Program description:** *SupportScreen*® completed by patients and CGs to identify biopsychosocial problems related to the cancer diagnosis. The electronic platform triages to professionals, provides tailored information and links to specialists +30-minute consultation with a joint male/female clinician-educators focused on sharing practical behaviours to help strengthen their relationship. Dyads are given educational handouts including practical suggestions. Clinician-educators consult privately with the surgeon to report demographics, support system, emotional regulation, screening results, and potential barriers to the treatment plan + consultation with surgeon who provided referrals as needed. **Control**: N/A **Duration:** Screening and one consultation **Intensity:** Minimum 30-minute consultation **Frequency of FROM screening:** Once (enrollment in study when patient contacts the team about scheduling a surgical consultation) **Clinician responding to FROMs:** Clinician-educators, the patient’s surgeon, and other clinicians upon referral **Alert system**: After the screening tool identified psychosocial distress, electronic communication ensures triage to clinicians	Compared to partners, patients reported significantly higher distress related to ‘feeling anxious or fearful,’ ‘managing my emotions,’ and ‘being unable to take care of myself.’ High distress areas for partners were most commonly ‘feeling anxious or fearful,’ ‘finances,’ ‘sleeping,’ and ‘feeling down or depressed.’ Mean satisfaction ratings of dyads (anonymous survey completed by 60 patients and 52 CGs) for the partners’ clinic ranged from 3.59/5 to 3.78/5 across survey items. The lowest rated item was information on the ‘partners guide to managing the challenges of breast cancer’ written worksheet ‘was useful;” and the highest was ‘I recommend this program, for other women and their partners.’
**11. Burridge et al, 2017**^35^ **Australia** (Related to Mitchell et al, 2013^49^)	Acceptability and feasibility study	To explore the appropriateness and utility of the needs assessment tool for caregivers (NAT-C), from the perspectives of CGs and general practitioners (GPs - also called family physicians), to help CGs address their health concerns with the support of their GP	**Setting:** Primary care **Population**: CGs of patients with advanced cancer **Sample:** N = 11 CGs **Mean age** (years): 58 (range = 32 -74) years **% female:** 63.6% **Race/ethnicity:** Not specified **Caregiver relationship**: 63.3% Spouse/partner **Patient illness:** Cancer **Duration of caregiving**: Not specified **Mean # of hours/week caregiving**: Not specified	Sub-study of Mitchell et al. (2013)^49^	1) Acceptability of the intervention: CGs found checklist simple and clear, but in terms of acceptability they had different perceptions about the importance of their own health and the relevance of discussing tool results with their GP. GPs found the tool acceptable but recognized the process could be onerous in a busy practice setting. 2) Impact of intervention on GP-CG relationship: Supportive skills of the GP and a well-established GP-CG relationship facilitated disclosing and detection of CGs’ outcomes and initiation of adequate support from the GP. 3) Place of intervention in advanced cancer care: GP support offered to CGs may be overlooked, while support available from cancer services and other clinicians may or may not be appropriate.
**12. Carduff et al, 2016** ^36^ **United Kingdom**	Acceptability and feasibility study	To develop, pilot, and assess a model for identifying, addressing, and supporting CGs of adults with palliative and supportive care needs in primary care	**Setting:** 4 general practices **Population**: CGs of people with palliative and supportive care needs (terminal illnesses) **Sample:** N = 11 CGs completed interviews **Mean age** (years): 74 (range 58-86 years) **% female:** 63.6% **Race/ethnicity:** Not specified **Caregiver relationship**: 72.8% Spouse/partner **Patient illness:** Terminal illnesses (dementia, lung disease, and cancer were most common) **Duration of caregiving**: Not specified **Mean # of hours/week caregiving**: Not specified	**Program description:** A CG liaison was nominated with each practice. CGs toolkit consisting of a cover letter + CSNAT + “who to call” fridge magnet. CGs who returned assessment packages were invited to conduct a follow up interview with the liaison person (face to face or telephone) who assessed CGs’ unmet needs by reviewing the completed CSNAT and offered relevant support. **Control**: N/A **Duration:** 12 months **Intensity:** Variable (completing CSNAT + variable length of follow-up conversation and use of support resources provided) **Frequency of FROM screening**: Once (baseline) **Clinician responding to FROMs:** Carer liaison (3 x clinical, 1 x administrative person) **Alert system**: Not specified	**Feasibility:** 81 CGs received the toolkit, of which 25 returned the completed CSNAT form. 20 of these CGs identified at least one unmet need, 18 had follow-up conversations with the interventionists, and 12 CGs were offered relevant support. Many CGs did not see themselves as ‘carers’ but rather viewed their role as a natural extension of their relationship with the patient and that some CGs were registered with a different practice than the patients and could not receive the intervention. **Acceptability** **CGs:** Endorsed the provision of available support through their GP practice but found that the paperwork was burdensome. CGs did not describe the need for intensive support, preferring smaller interventions. CG Liaisons and GPs agreed that it was important to identify and support CGs. Concerns included that it was difficult to identify CGs who might be struggling, and that support could be difficult to access/initiate for CGs (e.g., respite). GPs reported that greater awareness of available resources was needed.
**13. Darley et al,** **2020**^37^ **United Kingdom** (Related to Patchwood et al, 2021^52^)	Process analysis	To investigate factors impacting implementation and acceptability of the CSNAT-stroke to support CGs	**Setting:** Stroke organisation services across England and Northern Ireland **Population**: CGs of stroke survivors **Sample:** N = 21 CGs N = 31 staff members (frontline, managers, and leaders) completed interviews; 62 staff completed preintervention questionnaire **Mean age** (years): Not specified **% female:** 71.4% **Race/ethnicity:** Not specified **Caregiver relationship**: 66.7% spouse/partner **Patient illness:** Stroke **Duration of caregiving**: Not specified **Mean # of hours/week caregiving**: Not specified	**Program description:** CSNAT-stroke (see Aoun et al, 2015a^29^ for description of CSNAT approach) delivered typically at home visits. **Control**: Usual care **Duration:** Not specified **Intensity:** Not specified **Frequency of FROM screening:** Variable based on staff contact with CG; at minimum one face-to-face session with reviews likely **Clinician responding to FROMs:** Support coordinators (layperson) working for stroke organization **Alert system**: Not specified	Four major themes were identified: 1) **Coherence:** Staff suggested a limited understanding of the different components and stages of the intervention. 2) **Cognitive participation**: CG felt it was difficult to participate and get involve in the intervention. 3) **Collective action**: Some components of the intervention were not always implemented and administered by the frontline staff. 4) **Reflexive monitoring**: Staff felt it was difficult to share their learning and receive support from across the stroke organization.
**14. Edilloran et al, 2022** ^38^ **United States**	Quality improvement – pre-experimental (pre-post one group)	To improve support provided to CGs of inpatients hospitalized for stroke through tailoring services and education using a preparedness assessment tool	**Setting:** A comprehensive stroke center **Population**: Family CG of stroke survivors **Sample:** N = 19 CGs **Mean age** (years): 31.6% aged 60 + years **% female:** Not specified **Race/ethnicity:** Not specified **Caregiver relationship**: 47.4% Spouse/partner **Patient illness:** Stroke survivors with a primary diagnosis of ischemic stroke, hemorrhagic stroke, or transient ischemic attack **Duration of caregiving**: Not specified **Mean # of hours/week caregiving**: Not specified	**Program description:** Stroke survivor and CG support bundle intervention in which (1) CGs were asked to complete the preparedness for caregiving scale of the family Care Inventory; (2) CGs were provided with information on stroke, what to expect from hospital to home, CG burnout and local support/resources; and (3) individualized interventions from the care team based on the domains identified in the preparedness for caregiving scale as the CGs’ greatest needs. **Control**: N/A **Duration:** Variable - length of hospitalization in units. Study ran for 3 months **Intensity:** Not specified **Frequency of FROM screening:** Once (pre-intervention within 24 hours of admission) **Clinician responding to FROMs:** Multidisciplinary healthcare team consisting of physicians, nurse practitioners, nurses, (primary), rehabilitation therapists, social worker/case management **Alert system**: Not specified	Significant improvement in family CG readiness after exposure to the intervention. Pre-intervention needs assessment demonstrated stroke patients’ needs upon hospital discharge included education about mobility (85.18%); information about depression, anxiety, and coping skills (74.08%); activities of daily living (66.67%); and communication (62.97%) Patient satisfaction scores in communication, discharge, and care transitions were positively impacted during the project time frame.
**15. Ewing et al., 2016**^39^ **United Kingdom** (Related to Grande et al, 2017^40^)	Acceptability and feasibility study	To explore clinician perspectives of the role of the CSNAT to identify its impact and mechanisms of action in the context of palliative home care	**Setting:** Implementation first assessed in one small hospice at home service. This was followed by a feasibility study in a large hospice at home service. For contextual diversity, one setting was urban while the other rural, each differing in services size and staff composition **Population**: Staff members in the two settings **Sample:** N = 26 clincians (nurses, healthcare assistants)* Patient illness: Receiving palliative home care	**Program description:** See Aoun et al (2015a)^29^ for description of CSNAT approach. **Control**: N/A **Duration:** Not specified **Intensity:** Not specified **Frequency of FROM screening:** Not specified **Clinician responding to FROMs:** Varied based on palliative care site but included clinical nurse specialists, healthcare assistants, nurses and managers **Alert system**: Not specified	Clinicians who used the CSNAT within a CG-led process of assessment and support, identified positive impacts including providing greater visibility of support needs, legitimizing support for CGs and opening up different conversations with CGs. Creating a space for the separate needs of CGs, providing an opportunity for CGs to express support needs, and responding to CG-prioritized support needs were identified as mechanisms of action, enabling a positive impact when the CSNAT was used. Some of the difficulties clinicians describe in supporting CGs include CGs ‘not knowing’ what support might be available and not being aware of what they ‘don’t know.’
**16. Grande et al., 2015**^40^ **United Kingdom** (Related to Ewing et al, 2016^39^)	2-arm RCT (sub-group analysis of a randomized, stepped wedge cluster trial)	To evaluate the impact of the CSNAT intervention on CGs and patients during end-of-life care	**Setting:** Six palliative home care sites **Population**: CGs of patients with an end-of-life condition (mostly cancer diagnosis) who required palliative care and died during the trial. **Sample:** N = 681 CGs (T = 348; C = 333) **Mean age** (years): Not specified **% female:** T = 71%; C = 67% **Race/ethnicity:** Not specified **Caregiver relationship**: 67% Spouse/partner **Patient illness:** end of life condition (mostly cancer) **Duration of caregiving**: Not specified. **Mean # of hours/week caregiving**: Not specified	**Program description:** See Aoun et al (2015a)^29^ for description of CSNAT approach. **Control**: Usual care **Duration:** Not specified **Intensity:** Not specified **Frequency of FROM screening:** Not specified. At least once. **Clinician responding to FROMs:** Clinicians (varied based on palliative care site but included clinical nurse specialists, healthcare assistants, nurses and managers **Alert system**: Not specified	**Response rate:** There was no statistically significant effect for a difference in response rate across time/phase. **Outcomes:** T > C for reduction in early grief and improved mental and physical health. T = C for feeling their needs had been met (CSNAT total), or their support needs probed or listened to, to a greater extent. Evidence of a favourable intervention effect on probability of death at home and seeing the place of death as the best place. **Implementation of the intervention:** 985 CSNATs were handed out to CGs but CSNAT assessments were completed in only 209 (21.2%) of these cases (range = 13.7%-56.3%). CSNATs were reportedly handed out to 685 (41.6%) CGs of 1645 new patients and completed for 113 of these (completion rate 16.5%). Where total caseload numbers are available, CSNATs was handed out to 789 (32.1%) of 2461 on total case- load (completion rate 20.0%).
**17. Hawkes et al., 2010** ^41^ **Australia**	Acceptability and feasibility study	To examine the feasibility community-based helpline operators screening adults with cancer and their CGs for distresss and then triaging them to appropriate referral	**Setting:** Non-governmental charitable cancer helpline offering information and support **Population**: Adult CGs of people with cancer **Sample:** N = 118 CGs **Mean age** (years): Not specified (only presented combined patients) **% female:** 89.0% **Race/ethnicity:** Not specified **Caregiver relationship**: Not specified **Patient illness:** Cancer, most commonly lung (15.4%), liver (12.0%) or brain (8.5%) **Duration of caregiving**: Not specified **Mean # of hours/week caregiving**: Not specified	**Program description:** Systematic screening by cancer helpline operators (clinicians with oncology experience) using the DT and hospital anxiety and depression scale (HADS). Based on the scores and their clinical judgement, operators triaged participants to the appropriate level of care: - DT score 0-3: Supportive care (e.g., psychoeducation) - DT score 4-8: Extended care (e.g., coping skills training) or specialist care (skilled therapist for specific issue) - DT score 9-10: Acute care (specialist services or multidisciplinary team for broader issues) **Control**: N/A **Duration:** Not specified **Intensity:** Variable (initial call + variable intensity based on level of care/follow-up required) **Frequency of FROM screening:** Once (during initial call) **Clinician responding to FROMs:** Cancer helpline operators (clinicians with oncology experience) **Alert system**: Not specified	**Distress** Sensitivity and specificity maximized at cut off ^3^4 and ^3^6 for detecting general psychosocial morbidity (HADS^3^ 15), for patients and CGs, respectively. 72.8% of participants met^3^ 4 DT cutoff. CGs reported higher levels of distress than patients. Emotional problems were main reported cause of distress. Younger, female, and current employment associated with^3^ 4 DT (CGs and patients). **Anxiety and depression** 49.2% and 43.0% of CGs and patients, respectively, reached the HADS clinical cut-off ≥15. CGs more likely to be anxious than patients; no difference in depression status. **Triage to Tiered Care** 80.5% of callers (CGs and patients) were triaged to universal or supportive care; nearly 20% to extended care; one to specialist care; none to acute care. Patients reporting^3^ 4 DT were more likely to receive extended or specialist care; association between distress and level of care was not significant for CGs (caution due to small cell sizes).
**18. Heckel et al,** **2018a**^43^ **Australia** (Related to Heckel et al, 2018b^42^)	2-arm RCT	To evaluate the efficacy of a telephone outcall program (13 11 20 service) in improving CG burden, unmet needs, and psychological health among CGs of newly diagnosed cancer patients	**Setting:** Four outpatient oncology clinics **Population**: CGs of cancer patients **Sample:** N = 216 CGs (T = 108, C = 108) **Mean age** (years): 56.8 ± 12.9 **% female:** 57.4% **Race/ethnicity:** Not specified; 100% reported not being Indigenous (Aboriginal/Torres Strait Islander) **Caregiver relationship**: 79.2% spouse/partner **Patient illness:** Cancer (stages I-III) **Duration of caregiving**: Not specified **Mean # of hours/week caregiving**: Not specified	**Program description:** 13 11 20 outcall program involving: (1) 3 x telephone calls with an oncology nurse. Each phone call consisted of: (1) measuring CGs’ level of distress (DT); (2) addressing CGs’ unmet needs by raising six topics (psychological distress, health literacy, health, family support, financial, and practical issues); and (3) navigation to appropriate support services for follow-up care if required (defined as distress score ≥4 and an impact score ≥3). **Control**: Attention control group **Duration:** 4 months (consisting of 3 x phone calls) **Intensity:** 3 calls; mean of 22 min each **Frequency of FROM screening:** Three times (5-10 days post randomization, 1 month, and 4 months) **Clinician responding to FROMs:** Oncology nurse **Alert system**: Not specified	**Primary:** **T2 (1 months):** T = C for CG burden **T3 (6 months):** T = C for CG burden **Secondary:** **T1 (1 month):** T > C CG unmet needs. T = C for CG self- empowerment, health literacy, self-esteem and depression **T3 (6 months):** T = C for reduction of CG unmet needs, CG self-empowerment, health literacy, self-esteem and depression
**19. Heckel et al,** **2018b**^42^ **Australia** (Related to Heckel et al, 2018a^43^)	Acceptability and feasibility study	To evaluate the utility of a telephone outcall program for CGs of adults with cancer and to examine its impact on longitudinal changes in CGs’ supportive care needs and level of distress	**Setting:** Four outpatient clinics **Population**: CGs of cancer patients **Sample:** N = 216 CGs **Mean age** (years): Not specified, median = 59.5 (31 to 77) **% female:** 54.0% **Race/ethnicity:** Not specified **Caregiver relationship**: 79.0% spouse/partner **Patient illness:** Cancer **Duration of caregiving**: Not specified **Mean # of hours/week caregiving**: Not specified	See Heckel et al. (2018a)	**T3 (4 months):** T > C for CGs’ reported level of distress and impact of relative to T1 and T2 **Utility:** 95% of CGs felt it was worth their time, 82% found it relevant to them, 96% trusted the information provided, 96% found difficult topics and discussions were handled well. **Supportive care needs:** Psychological distress was the most frequently discussed topic (82%) by CGs, followed by health issues (51%), health literacy (45%), and family issues (44%). The frequency of psychological distress, health literacy, financial problems, and practical issues discussed by CGs decreased over time. Females were more likely to discuss psychological distress with the nurse and younger CGs (<55 years) were more likely to discuss family issues than older CGs.
**20. Kauffman et al, 2016** ^44^ **United States**	Acceptability and feasibility study acceptability study	To better understand the psychosocial needs of adults with breast cancer and their partners by examining their levels of psychosocial distress	**Setting:** Medical center **Population**: CGs of breast cancer patients **CG sample:** N = 82 CGs **Mean age** (years): Not specified **% female:** 23.3 **Race/ethnicity:** Not specified **Caregiver relationship**: 100% partner/spouse **Patient illness:** Breast cancer **Duration of caregiving**: Not specified **Mean # of hours/week caregiving**: Not specified	**Program description:** See Bitz et al. **Control**: N/A **Duration:** Not specified **Intensity:** Minimum 30-minute consultation **Frequency of FROM screening:** Once (baseline) **Clinician responding to FROMs:** Female and male social work team, the patient’s surgeon, and other clincians upon referral **Alert system**: After the screening tool identified psychosocial distress, electronic communication ensures triage to clinicians	Patients more likely than partners to report feeling fearful or anxious, difficulty in managing their emotions, experience distress about being unable to take of themselves, substance use by themselves or in their environment. No difference between patients and partners was found for feeling down or depressed, or in feeling unsupported by their partner.
**21. LeCouteur et al, 2022** ^45^ **Australia**	Acceptability and feasibility study	To investigate the utilization of the DT to screen patients and CGs by nurses working at a cancer-support helpline, and to examine how callers responded to this screening	**Setting**: Cancer helpline support for adult patients and their CGs **Population**: Nurses working in a cancer support helpline program **Sample:** N = 12 helpline nurses* Patient illness: Cancer	**Program description:** Nurses answered phone calls received at the cancer-support helpline; support was provided on a case-by-case basis (e.g. nutritional advice, physical symptoms, treatment regimens). Systematic screening of callers using the DT was performed. **Control**: N/A **Duration:** 1 x call of unknown duration; calls were retrieved for the data analysis over a period of 4 months **Intensity:** N/A **Frequency of FROM screening:** Once (during call) **Clinician responding to FROMs:** Nurses **Alert system**: Any caller reporting a score of ≥ 4 were referred to the organisation’s counselling service	40 calls conducted by nurses including 23 (57.5%) calls with patient and 17 calls with CG (42.5%) In 87.5% of calls, the DT screening was used at the end of the call and 12.5% in the middle. When brought up at the end, typically the DT was positioned in a closing sequence (series of institutional questions about demographics, etc.). In these cases, discussion about distress rarely continued beyond receiving the numerical score giving callers limited opportunity to elaborate on their distress. Referrals to psychosocial support were successful when the DT was brought up in the middle of calls (12.5% of calls). In these cases, nurses had oriented the call to include distress as a topic. Referrals were very rare when the DT was assessed at the end of a call. Nurses often distanced themselves from the DT using introductions like “this is just for stats.”
**22. Lund et al., 2020**^46^ **Denmark** (Related to Lund et al, 2022^47^)	2-arm RCT (stepped-wedge)	To evaluate the efficacy of the CSNAT-I intervention in improving strain, distress, fatigue, QoL, positive caregiving appraisals, experiences of the interaction with clinicians, workload, and emotional functioning of CGs of adults receiving specialized palliative care	**Setting:** Specialized palliative care (SPC) **Population**: Adult primary CGs of patients recently started in SPC **Sample**: N = 437 CGs (T = 182; C = 255) **Mean age** (years): 59.8 (Median = 61, 23.0 – 90.0) **% female**: 69.1% **Race/ethnicity:** Not specified **Caregiver relationship:** 61.7% spouse/partner **Patient illness:** Patients receiving specialized palliative care services related to a cancer dx (95%) or heart, lung, kidney or neurological diseases (5%) **Duration of caregiving:** Not specified **Mean # of hours/week caregiving:** Not specified	**Program description:** See Aoun et al (2015a)^29^ for description of CSNAT approach. The intervention, including conversation with member of the SPC team, was delivered twice. **Control:** Every cluster first acts as a control and later as an intervention site **Duration:** 1 month **Intensity:** 75 minutes (range 20 - 160 minutes) **Frequency of FROM screening:** Twice (within 13 days post-enrolment and 15-27 days post-enrolment) **Clinician responding to FROMs:** Member of SPC interdisciplinary teams comprised of specialised physicians, nurses, physiotherapists, social workers, psychologists or priests. Nurses were the predominant clinicians who carried out the intervention (91%–93% of conversations). **Alert system**: Not specified	**Primary**: **T1 (2 weeks):** T = C for CG strain **Secondary:** **T1 (2 weeks):** T > C for CG distress, home care responsibility, attention to the CGs’ wellbeing, quality of information and communication, amount of information, CG involvement, talking about greatest burdens, and assistance in managing greatest burdens **T2 (4 weeks):** T > C for attention to the CGs’ wellbeing, quality of information and communication, amount of information, CG involvement, talking about greatest burdens, and assistance in managing greatest burdens
**23. Lund et al., 2022**^47^ **Denmark** (Related to Lund 2020^46^)	Acceptability and feasibility study	From the perspectives of clinicians, to explore the process, content, and experiences of delivering the CSNAT-I intervention in SPC setting	**Same as Lund et al 2020** **Sample**: N = 130 CGs (subset of 437 who completed intervention in Lund et al 2020^46^) N = 44 clinicians who delivered intervention* CG demographics **Mean age** (years): 57.4 ± 13.0 **% female**: 75.0% **Race/ethnicity:** Not specified **Caregiver relationship:** 60.0% spouse/partner **Patient illness:** Patients receiving specialized palliative care services related to a cancer dx (96%) or heart, lung, kidney or neurological diseases (4%) **Duration of caregiving:** Not specified **Mean # of hours/week caregiving:** Not specified	**Same as Lund et al 2020** ^46^ **. See also Aoun et al (2015a)**	**Content of CSNAT-I:** **T1 (within 13 days of enrolment)**: CGs’ prioritised domains to discuss with clinicians were: 1) ‘knowing what to expect in the future’ (44%); 2) ‘dealing with feelings and worries’ (43%), and 3) ‘knowing who to contact if concerned’ (29%). **T2 (15-27 days after enrolment):** CGs’ most frequently prioritised domains were: 1) ‘dealing with feelings and worries’ (32%), 2) ‘knowing what to expect in the future’ (26%), and 3) ‘understanding the illness’ and ‘talking to the patient about the illness’ (both domains prioritised by 18%). 75% and 71% of action plans made in T1 and T2 respectively, required solely actions directly delivered by the clinician during the actual contact vs 8% and 17% of the action plans at T1 and T2 respectively required the clinician to take further action (e.g. referral). **Clinicians’** **experiences of CSNAT‐I:** Overall, clinicians found CSNAT-I as meaningful and constructive. Difficulties in delivery of CSNAT-I was rarely an issue.
**24. Marston et al., 2023** ^48^ **Australia**	Acceptability and feasibility study	To explore advanced cancer patients’ and their CGs’ perspectives on the acceptability of the CSNAT-I delivered during hospital discharge by occupational therapists	**Setting:** 140-Bed specialist cancer center and a 571-bed acute metropolitan hospital **Population**: CGs of adults with advanced cancer **Sample**: N = 10 CGs **Mean age** (years): 53.6 ± 15.0 **% female**: 70% **Race/ethnicity:** Not specified **Caregiver relationship:** 40.0% spouse/partner **Patient illness:** Advanced cancer, who were planning to return home and had complex discharge needs with occupational therapist referral **Duration of caregiving:** 31.8 months ± 47.6 **Mean # of hours/week caregiving:** Not specified	**Program description:** See Aoun et al (2015a)^29^ for description of CSNAT approach. Here, occupational therapists delivered the intervention. **Control:** N/A **Duration:** Variable **Intensity:** Not specified **Frequency of FROM screening:** Twice (Early admission and post-discharge) **Clinician responding to FROMs:** Occupational therapist **Alert system**: Not specified	Patients and CGs described CSNAT-I as a coordinated process enabling identification of CG discharge needs and practical ways to address them. Most CGs valued the structured approach to work through their needs, offering a smooth coordinated approach to an overwhelming situation. Checklist was easy to understand. Process of how these steps were enacted was not always clear to patients and CGs, particularly the process after discharge. CGs recalled this process in different ways, ranging from not knowing who should contact who to having contact with various clinicians involved in their care. CSNAT-I facilitated self-awareness of CGs’ own current and future care needs and priorities but also improved clinician awareness of CG needs. Confirming that there was help available that CGs would not otherwise know about and a way to acquire information. Enabled clinicians to gain immediate insights into what was needed, and CGs to better understand what areas clinicians were likely to be able to help with. CGs described feeling more supported, cared for, and reassured that they will be able to cope. CGs felt surrounded in this extra care that extended across hospital and home. CGs’ experiences of support from a coordinated team across hospital and community rather than a specific person.
**25. Mitchell et al, 2013**^49^ **Australia** (Related to Burridge et al, 2017^35^)	2-arm RCT	To evaluate the efficacy of a GP-based intervention in identifying and addressing CG unmet needs. The intervention included a CG-reported needs checklist and a supporting GP resource toolkit	**Setting:** Three oncology services + one palliative care service **Population:** Adult CGs of patients with advanced cancer **Sample:** N = 392 CGs (T = 194; C = 198) **Mean age** (years): 57.4 ± 12.81 % **female:** 66.5% **Race/ethnicity:** Not specified **Caregiver relationship**: 68% Spouse/partner **Patient illness:** Advanced cancer (locally invasive or metastatic disease) **Duration of caregiving:** Not specified **Mean # of hours/week caregiving**: Not specified	**Program description:** CGs completed the needs assessment tool - CGs (NAT-C) assessing their unmet needs across informational, physical, psychological, spiritual, existential, social, financial and legal domains. The CG booked a long appointment and presented for a NAT-C-guided consultation within 1 week of their GP being introduced to the study (baseline) and again at 3 months. GPs had no specific time limitation on the appointments and were encouraged to use the GP toolkit consisting of both paper-based and electronic materials organized into the domains in the NAT- C which provides links to evidence–based information, resources and services that might help address identified problems. **Control**: Usual care **Duration:** Guided consultations at baseline and 3-months after **Intensity:** 2 x long appointments (length unspecified) **Frequency of FROM screening:** Twice (baseline and 3 months) **Clinician responding to FROMs:** GPs **Alert system**: Not specified	**Primary:** **T1 (6 months):** T = C for number and intensity of unmet needs **Secondary:** **T1 (6 months):** T = C for anxiety and depression, and health related QoL at 6 months
**26. Norinder et al., 2022** ^61^ **Sweden**	Acceptability and feasibility study	To explore nurses’ experiences learning to use CSNAT-I intervention and supporting CGs in specialised home care	**Setting:** 6 specialized home care services in 3 geographic locations, including urban, suburban and rural areas **Population**: Nursing staff at these services **Sample:** N = 12 nurses* Patient illness: Life-threatening illnesses, including patients with palliative care needs	**Program description:** See Aoun et al (2015a) for description of CSNAT approach. Here delivered by nurses in specialized home care with continuous review of the action plan to identify changing CG needs. **Control**: N/A **Duration:** Not specificed. Interview data collected over 15-month period **Intensity:** 30-min training, resources provided, most nurses used the CSNAT-I at least twice **Frequency of FROM screening:** Not specified **Clinician responding to FROMs:** Nurses **Alert system**: Not specified	22 interviews conducted. Nurses’ everyday clinical practice changed while learning to use the CSNAT-I and they experienced professional and personal growth. Three key themes highlighted the changes in nurses’ ways of supporting family CGs: (1) from reactive towards proactive support; (2) From professional responsibility towards a shared responsibility; and (3) from ad hoc contacts in the hallway towards scheduled trustful conversations.
**27. Norinder et al., 2023a**^50^ **Sweden **(related to Norinder et al., 2023b^51^)	Pre-experimental (1 group pre/post design)	To investigate the potential effects of the CSNAT-I intervention on CG burden, QoL, and CG preparedness among spousal CGs of adults receiving specialized home care for life limiting illnesses	**Setting:** 6 specialized home care services in 3 geographic locations, including urban, suburban, and rural areas **Population**: Adult spousal CGs of palliative patients and patients with life-threatening illnesses receiving home care **Sample:** N = 33 CGs **Mean age** (years): 68.6 ± 8.3 **% female:** 57.6% **Race/ethnicity:** Not specified **Caregiver relationship**: 100% Spouse/partner **Patient illness:** Patients with life-threatening illnesses, including patients with palliative care needs. **Duration of caregiving**: Not specified **Mean # of hours/week caregiving**: Not specified	**Program description:** See Aoun et al (2015a) for description of CSNAT approach. **Control**: N/A **Duration:** Not specified. Completed the CSNAT-I at least once. Data collected baseline and 1.25 months **Intensity:** Not specified **Frequency of FROM screening:** Once **Clinician responding to FROMs:** Nurses **Alert system**: Not specified	**T1 (5 weeks):** CGs had significantly higher levels of preparedness and significantly lower levels of burden regarding environment. CGs had lower levels of general strain, disappointment, and emotional involvement that were not statistically significant. Likewise, higher level of isolation was not statistically significant. No change in QoL (descriptively or statistically)
**28. Norinder et al., 2023b**^51^ **Sweden **(related to Norinder et al., 2023a^50^)	Acceptability and feasibility study	To explore CG's experiences of the CSNAT-I intervention delivered by nurses in specialized home care	**Setting:** See Norinder et al., 2022^50^ **Population**: Adult spousal CGs of palliative patients & patients with life-threatening illnesses receiving home care **Sample:** N = 10 CGs (sampled from Norinder et al. (2023a)^50^) **Mean age** (yrs): Median age of 66 (39-75) **% female:** 90 **Race/ethnicity:** Not specified **Caregiver relationship**: 100% Partner/spouse **Patient illness:** Patients with life-threatening illnesses **Duration of caregiving**: Not specified. **Avg # of hours/week caregiving**: Not specified	**Program description:** See Norinder et al., 2022^50^ **Control**: N/A **Duration:** Not specified **Intensity:** Not specified **Frequency of FROM screening:** Variable. All participants had to have experienced the intervention at least once **Clinician responding to FROMs:** Nurses **Alert system**: Not specified	**Three themes identified:** **1) A chance to focus on what’s important.** Scheduled meeting indicated there was time to care for them as well and this opportunity was appreciated. Let them know what support was available. Questions were helpful starting point for discussion and help them decide what to prioritize. **2) Flexible and co-created dialogue.** CGs felt there was a shared responsibility with the nurse. However, some experienced it as being co-led, while others felt it was themselves or the nurse directing the discussion. The discussions were found to balance being focused and still open. **3) Finding solutions and gaining insights.** Some CGs felt that given the situations they were in, they did not often have time to think of their own needs. They also felt encouraged to take an active role addressing the issues they were facing, but felt the information and support offered by the nurses was helpful. For some, the conversations brought up difficult feelings about the future.
**29. Patchwood et al 2021**^52^ **United Kingdom** (Related to Darley et al, 2020^37^)	2-arm cluster RCT and economic evaluation	To examine the clinical and cost- effectiveness of the CSNAT-Stroke intervention for CGs of stroke survivors	**Setting:** Stroke organisation services across England and Northern Ireland **Population**: CGs of stroke survivors N **=** 414 CGs (T = 208; C = 206) **Mean age** (years): **62.4 (range 24-88)** **% female:** 77% **Race/ethnicity:** 97% white **Caregiver relationship**: 76% spouse/partner **Patient illness:** Stroke survivors **Duration of caregiving**: Participants were a mean 6-months post-stroke; 38.6% of CGs provided to survivor prior to stroke **Mean # of hours/week caregiving**: Not specified	**See Darley et al, 2020**	**Primary**:**T1 (3 months):** T = C for CG strain **T2 (6 months)**: T = C for CG strain **Secondary:** **T1 (3 months):** T = C for satisfaction with stroke services, anxiety and depression, service receipt inventory, health benefit **T2 (6 months)**: T = C for satisfaction with stroke services, anxiety and depression, service receipt inventory, health benefit Economic evaluation: No additional health benefits and slightly increased costs indicating the intervention is unlikely to be cost-effective.
**30. Philip et al., 2018** ^53^ **Australia**	Acceptability and feasibility study	To asessess the feasibility, acceptability, and preliminary effectiveness of I-CoPE (information, coordination, Preparation and Emotional support) for adults with high-grade glioma and their CGs	**Setting: N**euro-oncology service at a single tertiary center **Population**: CGs for newly diagnosed high-grade glioma patients that were planned for radiotherapy treatment. **Sample:** N = 31 CGs **Mean age** (years): 55.7 ± 12.9 **% female:** 58.1% **Race/ethnicity:** Not specified **Caregiver relationship**: 80.7 % Spouse/partner **Patient illness:** Cancer (Grade 3 or 4 glioma) **Duration of caregiving**: Not specified - newly diagnosed patients **Mean # of hours/week caregiving**: Not specified	**Program description:** Provision of I-CoPE intervention for patients and CGs, delivered by a cancer care coordinator across 3 key identified care transitions: (1) face-to-face consultation following the patient’s diagnosis in which the dyad received a resource folder including general (e.g. information about high-grade gliomas, treatment options) and tailored (e.g. patient’s diagnosis, site of tumor, some potential effects of a tumor in that specific site) information; (2) telephone follow-up 7 - 10 days post-discharge; and (3) either a face-to-face or telephone follow up after completion of radiotherapy in which the dyad received information package tailored to their needs, plus end-of-treatment information, community links, and follow-up information. Screening with DT and supportive counseling by the cancer care coordinator occurred at each of the three contacts + referrals to psychological support services as needed. **Control**: N/A **Duration:** Variable; delivered at 3 transition time points **Intensity: Mean** 69 minutes (range = 45-130) per CG + spontaenous calls to care coordinator (median 2, range 0-11) **Frequency of FROM screening:** Three times at transition points (baseline – following diagnosis after biopsy/resection in acute setting, 7-10 days post-discharge from acute setting, and following completion of standard radiotherapy protocol) **Clinician responding to FROMs:** Cancer care coordinator (nurse with oncology experience) **Alert system**: Not specified	High feasibility - 96% enrollment rate; completion rate of 94% among CGs. High acceptability among CGs as 87% rated the care received as “excellent.” The intervention significantly reduced supportive care needs and the need for information and increased the CG’s preparedness for care. There was no substantial change in QoL.
**31. Seipp et al., 2022** ^12^ **Germany**	Acceptability and feasibility study	To explore how patient-reported outcome measures [integrated palliative Care Outcome scale (IPOS) and IPOS views on Care (IPOS VoC)] and FROMs [Zarit burden Interview-7 items (ZBI-7)] can be feasibility, acceptably, and appropriately implemented in the daily care of healthcare teams providing specialized outpatient palliative care	**Setting:** Outpatient palliative care **Population**: Five outpatient palliative care teams (∼20 team members each; primarily nurses and physicians) **Sample:** N = 14 clinicians participated in focus groups (a greater number were trained and used the measures in practice)* Patient illness: Mostly cancer patients	**Program description:** Team members provided paper or software versions of the measures to patients and their CGs and collected these once complete. **Control**: N/A **Duration:** 11 months (study length) **Intensity:** N/A **Frequency of FROM screening:** Twice (on admission and at least once during further care) **Clinician responding to FROMs:** Outpatient, multidisciplinary palliative care teams (primarily nurses and physicians) **Alert system**: Not specified	ZBI-7 (FROM) use rejected by clinicians. They found that relatives were outraged by questions on whether providing care had caused them to lose control of their lives, as CGs felt caring is a natural duty that they wanted to fulfill clinicians thought using the measure was unsuitable because family CGs’ answers could burden the patients (e.g. when they are asked whether the patient affected ‘relationships with other family members/friends in a negative way’). They also criticized the measure for not providing the differentiated feedback that would be helpful in practical work. Researchers eventually removed ZBI-7 from the set of measures. Instead, they developed and implemented a questionnaire for CGs based on patient version of the IPOS VoC.
**32. Shaffer et al, 2019** ^54^ **United States**	Acceptability and feasibility study	To examine the feasibility and acceptability of the cancer support Source-CG tool from the perspectives of CGs of people receiving care at an ambulatory cancer surgery centre	**Setting:** Ambulatory cancer surgery center **Population**: CGs presenting with a patient with cancer **Sample:** N = 17 CGs **Mean age** (years): 59 (36-83) **% female:** 53% **Race/ethnicity:** Not specified **Caregiver relationship**: 76.5% Spouse/partner **Patient illness:** Cancer (breast, prostate, thyroid) **Duration of caregiving**: Not specified **Mean # of hours/week caregiving**: Not specified	**Program description:** Using a tablet, CGs were given the cancer support Source-CG tool which screens CGs for level of concern for 33 potential problems (self-care needs, emotional well-being, caregiving tasks, and patient’s well-being) and they would like concerns addressed. Based on level of concern for each problem, they were offered educational material and/or referral request or that no action be taken. **Control**: N/A **Duration:** 1 session **Intensity:** Mean 11.3 minutes (6-18) to complete the screening **Frequency of FROM screening:** Once **(**baseline) **Clinician responding to FROMs:** Hard copies of materials and information about requested materials provided by the study team **Alert system**: CGs who met criteria for depression risk were assessed for imminent self-harm and provided with a referral to the hospital’s counseling center.	**Overall distress score:** 31.1 ± 26.8 (range: 0 to 132). **Depression risk:** 5/17 CGs met the depression risk threshold score of 5 or greater. **Information needs:** 94.1% of CGs reported that at least one item was of moderate or greater concern (median = 8, range = 1-31 items of moderate or greater concern per person). Leading concerns were recurrence of cancer, the future, exercising and being physically active, and CGs’ own health needs. 10/17 CGs requested informational resources for at least one concern area. Leading information needs were patient well-being, exercising and being physically active, and managing symptoms and side effects of treatments.
**33. Sterba et al., 2017** ^56^ **United States**	Acceptability and feasibility study	To develop and pretest a tablet-based survivorship needs assessment planning tool (SNAP) and generate tailored plans to prepare dyads of patients and CGs for the post-treatment period	**Setting:** Outpatient (a single care center) **Population**: Primary CGs of head and neck cancer survivors **Sample:** N = 14 dyads (survivors + CG) N = 10 clinicians* Mean age (years): CGs: 61 (range = 29-83) **% female:** CGs: 57.0% **Race/ethnicity:** CGs: 93% European American **Caregiver relationship**: 71.00% Spouse/partner **Patient illness:** Head and neck cancer survivors within the past 2 years **Duration of caregiving**: 41% completed treatment within the last 12 months; and 59% within 13-24 months **Mean # of hours/week caregiving**: Not specified	**Program description:** Dyads completed a mock SNAP assessment via touchscreen tablet computer. SNAP includes an administrator planning interface (including developing libraries of referrals and educational material), data collection application, and tailored algorithm-driving care report. The assessment consisted of 10 questions concerning symptoms, unmet needs, and health behaviors + review of a mock written care plan including an example head and neck cancer survivor’s clinical characteristics followed by 3 sections with messages, referrals, and educational materials. **Control**: N/A **Duration:** 1 x session **Intensity:** Pre-test took place as part of an interview lasting approximately 45-60 minutes **Frequency of FROM screening:** Once (baseline, during interview) **Clinician responding to FROMs:** N/A **Alert system**: Not specified	**High acceptability:** CGs were comfortable holding tablets (77%), using tablets to read (92%) and answer (85%) questions. They reported high comfort in following instructions for questions (92%) and moving from one question to another (85% CGs). Most were in strong agreement that the information provided in the care plan was helpful emotionally (92%) and practically (92%).clinicians reported favorable impressions of the written care plan but highlighted time and clinic space availability as barriers to SNAP visit implementation. **Timing:** Clinicians recommended 1-6 months after treatment for survivorship visits, but CGs preferred earlier visits.
**34. Sterba et al., 2019** ^55^ **United States**	Pre-experimental (1-group pre-post)	To 1) assess the feasibility and acceptability of the SNAP intervention for head and neck cancer survivors and their CGs, 2) examine the short-term changes in their psychosocial outcomes having compled the intervention, and 3) refine the intervention for future use	**Setting:** Cancer center **Population**: Head and neck cancer survivors and their primary CGs **Sample:** N = 29 dyads (survivors + CG) N = 12 clinicians* CG demographics: **Mean age** (years): Not specified. Median = 56 (33-75) **% female:** 73.0% **Race/ethnicity:** 85% white, 12% African American, 4% Asian **CG relationship**: 77.0% Spouse/partner **Patient illness:** Head and neck cancer survivors who completed all treatment for stage I–IVA cancer of the upper aerodigestive tract (lip/oral cavity, pharynx, larynx, salivary gland, paranasal sinus) **Duration of caregiving**: Not specified **Mean # of hours/week caregiving**: Not specified	**Program description:** SNAP intervention consisting of: (1) SNAP clinical visit during which dyads completed, by tablet, separate questionnaires on psychosocial needs including unmet needs, dyadic efficacy, and distress and burden for CGs; (2) reviewed an algorithm-driven tailored care plan including messages, referrals, and educational materials with a nurse practitioner, who also answered participants’ questions, finalized the referrals and delivered a final printed care plan and tailored resource binder. **Control**: N/A **Duration:** 1 session **Intensity:** Mean 6 min for CGs and 11 min for survivors to complete tablet survey + mean 13 min discussion with nurse practitioner **Frequency of FROM screening:** Once (during SNAP clinic visit) **Clinician responding to FROMs:** Nurse practitioner **Alert system**: Not specified	**Primary:** **T (6 weeks):** T > C for depression, unmet needs, survivorship knowledge for CGs and survivors T > C for CG burden T = C for dyadic coping for CGs and survivors **Secondary** **T (6 weeks):** T > C for symptom distress and symptom management abilities for CG T > C for symptom distress and symptom management abilities for survivors T = C dyadic efficacy, follow-up care satisfaction, follow-up care adherence self-efficacy, and efficacy to obtain cancer related advice for CGs and survivors **Acceptability and feasibility:** Impact of the SNAP session 6-weeks later: Moderate-to-strong agreement 80%–100% on all items for CGs and survivors. Some CGs were highly interested in receiving a private visit to assess their concerns, and 56% preferred a session earlier in care. Dyads recommended further supportive care information. Dyads and clinician feedback revealed 3 themes on the SNAP sessions: (1) pulled together complex clinical information and facilitated care coordination, (2) offered continued support and addressed post-treatment needs, and (3) confirmed importance of CG well-being and dyad communication.
**35. Thomsen et al., 2017** ^57^ **Denmark**	Acceptability and feasibility study	To assess the feasibility of an intervention for CGs of adults receiving specialized palliative care. The intervention included a risk and needs assessment and tailored support from the interdisciplinary team	**Setting:** Outpatient, palliative care service **opulation**: CGs for patients in palliative services **Sample:** N = 76 CGs **Mean age** (years): Not specified **% female:** Not specified **Race/ethnicity:** Not specified **Caregiver relationship**: Not specified **Patient illness: Not** specified **Duration of caregiving**: Not specified **Mean # of hours/week caregiving**: Not specified	**Program description:** Intervention consisting of: (1) a home visit during which CGs complete a risk and needs assessment; (2) CGs risk factors and support needs were then discussed at an interdisciplinary conference, and a support plan was created; (3) targeted support was provided to CGs including a formalised supportive caregiver session with a nurse, a psychologist, a chaplain, volunteer support or referral to the GP to assess specific issues (e.g. depression); (4) support was documented in the CGs electronic medical record (which was put in place). **Control**: N/A **Duration:** Not specified **Intensity:** Not specified **Frequency of FROM screening:** Once (during 1^st^ home visit) **Clinician responding to FROMs:** Interdisciplinary team **Alert system**: Not specified	**Fidelity:** 75% of included CGs had a risk and needs assessment discussed at the interdisciplinary conference and had a targeted support plan with follow-ups. 62% of CGs who received targeted support had the results documented in their electronic medical record. **Acceptability:** Clinicians reported a reluctance to introduce the risk and needs assessment form to CGs due to CGs’ elicited emotional responses and clinicians feeling underprepared to intervene at the first home visit. Clinicians also reported a shortage of time to discuss CG concerns at the interdisciplinary conference. Clinicians agreed that the intervention provided an overview of the CGs needs and what to ask during next encounter.
**36. Toye et al., 2016** ^58^ **Australia**	2-arm RCT	To evaluate whether the Enabling Care at Home intervention delivered to CGs of adults discharged from an acute medical unit positively influenced CG, patient, and healthcare system outcomes. The intervention included telephone support and CG needs assessment delivered by a nurse	**Setting:** Medical assessment unit **Population**: CGs **Sample:** N = 175 dyads (T = 86; C = 89) **Mean age** (years): 62.1 ± 13.0 **% female:** 73.8% **Race/ethnicity:** Not specified **Caregiver relationship**: 29.8% Spouse/partner **Patient illness:** Patient aged 70 years or older being discharged to their home from a medical assessment unit for various medical illnesses **Duration of caregiving**: >24 months n = 99 (70.2%) **Mean # of hours/week caregiving**: Not specified	**Program description:** Usual discharge care plus the *Further Enabling Care at Home* program, which involved the implementation of three telephone calls made by a trained nurse, which included support to enhance understanding of the patient’s discharge letter, needs assessment with the CSNAT (see Aoun et al. (2015a)^29^ **Control**: Usual discharge care **Duration: ˜ **14 days **Intensity:** 3 phone contacts within approximately 14 days post discharge **Frequency of FROM screening:** Once (CSNAT provided at first contact, assessment conducted during second phone call, and review of action plan during the third call) **Clinician responding to FROMs:** Nurses **Alert system**: Not specified	**Primary:** **T2 (15-21 days post-discharge):** T > C for preparedness for caregiving across 5 domains (physical care, service finding/set-up, stress of CG; getting support; and overall preparedness) as well as mean overall total score **T3 (6 weeks post-discharge):** T > C for preparedness for caregiving across 3 domains (service finding/set-up, stress of CG; getting support) as well as mean overall total score **Secondary:** **T2 (15-21 days post-discharge):** T > C for greater improvement in overall symptoms, decreased CG distress. T = C for CG strain **T3 (6 weeks post-discharge):** T > C for greater improvement in overall symptoms, decreased CG strain. T = C for CG distress
**37. Wishart et al, 2020** ^59^ **Australia**	Observational cohort study	To assess the prevalence and nature of general and mealtime distress in CGs of adults with head and neck cancer using an eFROM system, *ScreenIT Carer, *as well as investigate factors associated with severity of distress	**Setting:** A quaternary hospital and cancer center **Population**: CGs of patients with head and neck cancer receiving curative radiation therapy **Sample:** N = 135 CGs **Mean age** (years): Not specified **% female:** 77.0% **Race/ethnicity:** Not specified **Caregiver relationship**: 62.2% Spouse/partner **Patient illness:** Head and neck cancer **Duration of caregiving**: Not specified **Mean # of hours/week caregiving**: Not specified	**Program description:** *ScreenIT Carer* is a computerized screening system in which CGs complete basic demographic information, general distress screening with the DT, mealtime-specific distress with a modified version of the DT, and need for supportive care services. *ScreenIT Carer* was accessed via secure login either in the waiting area, or from home. Summary reports of current responses were generated for clinicians. Risk management algorithms assessed the need for supportive care services and referral pathways to the multidisciplinary team. **Control**: N/A **Duration:** CGs encouraged to complete screening from planning until 2 weeks post curative radiation therapy (at patient appointments – no rules regarding mandatory attendance of CGs at these appointments) **Intensity:** Screening took approximately 2 minutes for participants to complete. Time dedicated to follow-up not specified **Frequency of FROM screening: Variable. With the aim of screening w**eekly **Clinician responding to FROMs:** “Clinicians” not otherwise specified **Alert system**: Referral pathways to the team based on risk management algorithms were actioned based on responses. Primarily social workers alerted to urgent/high risk responses and **provided** an assessment and directed to appropriate resources. Nurses also alerted.Alters received via email from the platform.	On average, CGs completed *ScreenIT Carer* 3 times over the course of radiation therapy (range 1-10) 58% and 46% of CGs experienced clinically significant levels of general distress and mealtime-specific distress, respectively. CG distress is associated with geographical location, treatment type, tumor site. Adherence was 41% (range = 11%–100%). Only 18% of CGs completed >80% completion of the *ScreenIT Carer* entries.
**38. Zapata et al., 2023** ^60^ **United States**	Acceptability and feasibility study	To develop a CG needs assessment tool and CG toolkit as well as to collect feedback from pilot implementation in outpatient palliative care sites to help establish lessons learned to support future dissemination	**Setting:** Seven outpatient palliative care teams providing home-based and clinic-based palliative care and both disease-specific and disease-agnostic services **Population**: CGs of patients receiving palliative care **Sample:** N = 339 CGs **Mean age** (years): 67% aged 50-74 **% female:** 70.0 % **Race/ethnicity:** 70.29% white, non-Hispanic; 14% Hispanic; 9% Asian, 3% Black/African American (all others 1%) **Caregiver relationship**: 57% Spouse/partner **Patient illness:** Those receiving palliative services from these teams **Duration of caregiving**: Not specified **Mean # of hours/day caregiving**: 32% < 5 hours/day; 31% 5-15 hours/day; 37% 15+ hours/day caregiving	**Program description:** Assess CG distress, well-being, and unmet needs as well as provision of CG toolkit, which included information on what to expect as a CG, navigating the healthcare system, talking with children about serious illness, finances, advance care planning, what to expect at the end of life, self-care information, and CG resources specific to the illness of their care recipient. For accessibility, abbreviated and more detailed components on each topic included in the toolkit.. **Control**: N/A **Duration:** Not specified **Intensity:** Not specified **Frequency of FROM screening:** Twice (baseline and follow-up) **Clinician responding to FROMs:** Clinicians from two or more disciplines (medicine, nursing, social work, chaplaincy, and/or administration) **Alert system**: Not specified	CGs reported clinically significant distress in 69% of cases (defined as DT score >4). Emotional concerns were among CGs’ most unmet needs, namely fear/worry/anxiety (57%), feeling overwhelmed/exhausted (51%), sadness (41%) and anger/frustration (39%). CGs rated the overall toolkit usefulness 8.4/10, SD = 1.8, easy to read, comprehensive, and helped them feel supported. Clinicians reported the biggest barrier to integration of the needs assessment into their workflow was time.

***Notes*.** *Only sociodemographic information on caregiver participants reported in Table 1.

^a^The CSNAT has been updated to include 15, rather than 14 domains, and the tool is used as part of a 5-stage intervention process (CSNAT-Intervention).^62^ https://csnat.org/. Click or tap if you trust this link.”>https://csnat.org

Abbreviations: T, treatment group; C, control group; T, C, no significant difference between treatment and control; T > C, treatment on reported outcome greater or higher than control; T < C, treatment on outcome lower or less than control. CG, caregivers; CSNAT, Carer support needs assessment tool (intervention); CSS-CG, CancerSupportSource^TM^-Caregiver; DT, distress thermometer; eFROM, electronic family-reported outcome measure; FROM, family-reported outcome measure; GP, general practitioner (also called family physician); HADS, Hospital Anxiety and Depression Scale; I-CoPE, Information, Coordination, Preparation and Emotional support; IPOS, Integrated Palliative Care Outcome Scale; IPOS VoC, Integrated Palliative Care Outcome Scale - Views on Care; IQR, inter-quartile range; MSK, Memorial Sloan Kettering Cancer Center; N/A, not applicable; NAT-C, Needs Assessment Tool for Caregivers; PROM, patient-reported outcome measure; QOL, quality of life; RCT, randomized controlled trial; SD, Standard deviation; SNAP, Survivorship needs assessment planning tool; SPC, Specialized palliative care; ZBI-7, Zarit Burden Interview-7 items.

#### Study Designs

Designs included 9 RCTs,^31-33,40,43,46,49,52,58^ 2 quasi-experimental studies,^28,29^ 3 pre-experimental studies,^38,50,55^ 22 acceptability and feasibility studies,^12,25-27,30,34-36,39,41,42,44,45,47,48,51,53,54,56,57,60,61^ 1 process analysis,^37^ and 1 observational cohort study.^59^

#### Participants

Across studies, 33 included non-duplicate samples (remaining were sub-studies). Specifically, 14 studies reported on only caregivers,^29-32,36,38,40,46,49-52,54,59^ 8 included caregivers and care recipients,^33,34,41,43,44,48,53,58^ 4 caregivers and clinicians/clinic staff (e.g., managers),^27,37,57,60^ 4 only clinicians/clinic staff,^12,26,39,61^ and 3 included caregivers, care recipients, and clinicians.^45,55,56^ Across these studies, 4312 caregivers were included (range = 10 to 681). Where reported, ages ranged from 22-90 years old. Most caregivers were female spouses of the care recipients. Most commonly, caregivers were providing care to someone with cancer^31,32,34,41,43,44,48,49,53-56,59^ or receiving palliative care (mixed diagnoses, but often cancer).^29,36,39,40,46,50,51,57,60^ The remaining studies included caregivers of care recipients who had a stroke,^38,52^ or adults experiencing motor neurone disease,^27^ dementia^30^ schizophrenia,^33^ and older adults hospitalized on a general medical assessment unit.^58^

#### Caregiving Context

Most studies did not provide information on caregiving context. In studies reporting mean duration of care, it ranged from 20.8 months^29^ to 49.0 months.^30^ The hours spent on caregiving^33,60^ ranged from <5 hours to 44 hours per week. One study^30^ reported that 66.6% of caregivers cared for one person. Similarly, two studies reported that caregivers were the sole person providing care to the care recipient.^30,59^ Most caregivers (81.6%) lived in the same household as the care recipient.^30,32,49,50,52,53,58^

### FROM Programs

#### FROM Program Settings

More than half of FROM programs were delivered in cancer centers^31,32,34,44,48,53-56,59^ or home-based palliative care/care for those with life threatening illnesses and complex care needs.^12,29,40,46,50,51,57,60,61^ Other settings included phone helplines,^41,43,45^ community care organizations,^27,30^ an inpatient general medical unit,^58^ primary care practices,^36,49^ a hospital stroke center,^38^ primarily home-based specialist stroke services,^52^ or by a family support worker in collaboration with a clinical psychologist on the study team in participants' homes.^33^

#### FROM screening

##### Types of FROMs

Only one study included a FROM for physical symptoms (Edmonton Symptom Assessment System)^32^; otherwise, all FROMs related to unmet needs, distress, burden, or psychological concerns (stressors, anxiety, depression, emotional well-being). All but 6 studies^25-36,39,40,43,44,46-52,54-58,60^ screened for unmet needs, concerns, or stressors. Over half screened using a single measure, with most screening for unmet needs or stressors.^12,27,29,30,33,34,36,40,44,46,48-52,58,61^ Fewer studies used 2-3 FROMs, which, aside from unmet needs, most commonly assessed distress.^32,43,45,54,59,60^

Common unmet needs measures were the Carer Support Needs Assessment Tool (CSNAT)^27,29,30,36,40,46,48,50-52,58,61^ (used as part of a 5-stage intervention process)^62^ or the Needs Assessment Tool for Caregiver (NAT-C).^35,49^ The most common measure related to distress, burden, anxiety, or depression was the Distress Thermometer (DT).^32,41,43,45,53,59,60^ In few studies, care recipients also completed equivalent PROMs, these included the DT,^41,53^ a biopsychosocial stressors related to breast cancer,^34,44^ and screening of near end-of-cancer-treatment emotional and physical challenges (e.g., unmet needs, symptom severity, fear of recurrence).^55,56^

##### Timing of FROMs

Most often FROMs were administered once (n = 11) to tailor an intervention/clinical response.^31,34,36,38,39,44,45,50,55,57,58^ Seven studies tailored their interventions by screening^27,30,35,46,48,49,60^ once before the intervention/clinical response and a second time at a follow-up assessment. Six studies included a varied number of screenings based on scheduled treatment or appointments with clinicians.^12,32,40,51,52,59^

##### FROM Administration

Most commonly, pen-and-paper versions of FROMs were used,^27,30,32,36,38,40,48,49,57^ followed by electronic and web-based platforms,^31,34,44,54-56,59^ and one used both paper and electronic formats.^12^ In the remaining studies, the format was unclear.^27,29,41,43,45,46,50-53,58,60^

#### Communication of FROM Screening Results

None of the programs reported providing summary reports aggregating the caregivers’ responses over time to either the interventionists or the caregivers. Rather, it was the responsibility of those in one particular clinical role, typically nurses, to interpret the FROM data and respond.^29,32,43,45,50,51,53,55,58,61^ In 8 programs, various members of the interdisciplinary team were expected to review the FROM data and respond.^12,27,38,40,44,46,57,60^

#### Response to FROM Screening

##### FROMs Clinical Follow-Up

In response to the FROMs, caregivers were: (a) provided with tailored information/educational resources that caregivers were expected to read on their own^27,29-32,34,36,38,40,41,44-46,48-55,58,60,61^; (b) referred to clinicians (e.g., psychologist) or community services^27,29-32,34,36,38,40,41,44-46,48-54,57-59,61^; and/or (c) offered individualized real-time feedback sessions with the interventionist(s) responsible for responding to the FROM data.^27,29-32,34,36,40,43,45,46,48-53,55,57,58,60,61^ Most programs included all three.

In addition to this follow-up, two programs provided more intensive support including communication-based problem-solving^34,44^ and counseling with psychoeducation and coping skills training.^41^ Twelve programs described developing caregiver care plans based on the FROMs.^27,29,30,36,40,46,48,50-52,55,56,58^

##### FROM-Based Severity Triage

All programs tailored their clinical follow-up based on the FROM results, but a severity triage was used in four programs that screened for distress and/or depression.^31,41,54,59^ In the study by Hawkes et al.,^41^ helpline operators used a stepped-care approach based on caregivers’ level of distress. In addition to basic information and advice, psychoeducation and emotional support were provided for DT scores of 0-3, more focused psychoeducation and coping skills training with a therapist were offered for scores of 4-8, and more acute care was offered for scores of 9-10, including specialist services or multi-disciplinary team intervention. In two studies,^31,54^ caregivers were offered education materials for items rated as being of lower severity; they were additionally offered the option of speaking with someone (e.g., referral) for higher severity concerns. Shaffer et al.^54^ noted that while referrals were offered based on the severity of the concern, caregivers had the option of approving or refusing the referral.

Four programs described responses to possible emergencies. All these programs included screening for distress and/or depression. In two studies,^31,54^ caregivers who met the criteria for depression were assessed for self-harm and provided a referral for counseling. In a helpline-based FROM program,^45^ callers reporting a score of 4 or more on the DT were referred to the organization’s counselling services, and in another study^32^ the nurse liaised with the caregivers’ family physicians when high distress was reported.

### FROM Adherence

Only two studies^32,59^ explicitly reported on caregivers’ adherence to completing the FROMs. In the study by Wishart et al.,^59^ 135 caregivers were asked to complete electronic FROMs weekly during the care recipients’ cancer treatment. Overall, the mean adherence rate was 41% (range 11-100%). Full adherence (defined as ≥ 80% of FROMs completed) was achieved by 18% of caregivers, 32% were partially adherent (33-79% completed), and 50% demonstrated low adherence (< 33% completed). However, attendance at the appointments was not mandatory so screenings may have been missed because the caregiver was not present. The 54 caregivers who participated in the intervention arm of an RCT^32^ were asked to complete distress screening every two months for up to 9-months. This was followed by a meeting with an oncology nurse who liaised with their family physicians, if distress was high. All caregivers completed baseline screening and an initial 20-minute meeting with a nurse. Adherence to the subsequent FROMs was 83%. The number of contacts with the oncology nurse was not specified; however, it was noted that though the nurse was made available for contact, only two caregivers reached out, and the nurse initiated all other contacts. In both studies, the FROM screenings were estimated to take less than three minutes to complete.

## Clinician and Caregiver Feedback

### Clinician Feedback

Fourteen studies reported on clinicians’ views of the FROM programs, with most (n = 9) analyzing qualitative data.^12,36,39,47,53,55,56,60,61^ Feedback was predominantly positive, with clinicians lauding the inclusion of caregivers in care^12,36^ and the structured method through which to support caregivers.^26,37,53^

#### Perceived Benefits Improved communication

Improved communication with caregivers was reported.^26,39,47,53,57^ The programs fostered conversations that might not have occurred otherwise,^26,39,47,61^ enabled discussions on challenging topics,^53^ and guided clinicians on what questions to ask.^57^ FROM programs also helped identify issues clinicians had not anticipated, challenged assumptions about which caregivers required support,^26,27,39,61^ and strengthened caregiver-clinician relationships.^26,61^

##### Focus on Caregivers’ Needs

FROM programs were seen as legitimizing caregivers’ needs, providing implicit “permission” for caregivers to request support.^12,26,27,39^ Formal screenings were noted as legitimizing clinicians’ spending clinical time on caregivers^26^ and making caregivers more visible in their work.^61^ Acknowledging caregivers’ needs was seen in itself as therapeutic.^27,60^

##### Increased Efficiency

Clinicians noted that FROM programs could streamline resource allocation through the early identification of concerns,^26,39,53,57,61^ receiving fewer spontaneous calls from care recipients and caregivers,^53^ and by reducing resource use for more problematic concerns later in the illness trajectory.^57^

#### Perceived Barriers

##### Concern about Responding to Results

Some clinicians were reluctant to administer the FROMs due to concerns that it could feel awkward or raise issues they are unprepared for,^26,57^ or lack the resources to address.^36,37,47,61^ FROM screening might unrealistically raise expectations of available services.^26,39^ Across studies, clinicians also reported the challenge of balancing a focus on caregivers with the care needs of care recipients.^27,47,57^ In one study, nurses were more likely to endorse actions/solutions related to supporting the caregiver in caring for the care recipient directly (e.g., information about the care recipient's medication) as being within their purview rather than supporting the caregiver in meeting their own needs (e.g., the caregiver’s health).^37^

##### Increased Workload

Clinicians suggested several ways that increased workload could potentially be attenuated,^12,26,37,47,55-57^ including recognizing caregivers as being clients, which would allow for corresponding time and resources dedicated to meeting their needs as well as the care recipient’s,^26^ limiting required documentation,^26^ ensuring it did not duplicate existing practices,^37^ integrating screening and response into their existing notes,^26^ and assistance of administrative team members, lay navigators, or social workers in coordination (e.g., follow-up reminder calls to caregivers).^60^

##### Added Burden for Caregivers

Clinicians also noted apprehension about FROMs adding to caregivers’ burden^26,39,47^ and/or potentially increasing their distress or anxiety.^26,39^ They articulated worry about caregiver readiness to discuss certain issues^39^ or the screening unearthing potential problems caregivers had not anticipated.^26^

#### Structural Barriers

These barriers included no clear guidelines regarding whose role it was to respond to caregiver needs,^56,57^ limited contact with caregivers in the clinic,^60^ a lack of physical clinic space,^55,56^ difficulty speaking with caregivers alone (and whether this was allowed),^37,47^ and insufficient time to discuss caregivers at team meetings.^57^ The need for further training to undertake screening and response was also emphasized,^26^ as were ethical concerns regarding creating a medical record for caregivers who did not have a formal diagnosis^57^ as well as what information could be shared with caregivers if the care recipient was not present.^47^

#### Perceived Facilitators

Flexibility in when and how the FROM screening was undertaken was a facilitator.^12,26,27,39,56,57,60^ Clinicians preferred that screening not occur at predefined times but, rather, when they felt it was appropriate^12,27,39^ or as needs arose.^39,56,60^ Clinicians reported developing their own ways of introducing the FROMs,^39^ including adapting the questions to the individual rather than reading word for word.^12,61^ The option of remote or telephone screening was also highlighted as a potential facilitator.^53,55^ Screening for outcomes that clinicians felt were relevant and ensuring that clinicians understood how the results would be used were perceived as critical to their acceptance.^12^ Clinicians reported that settings in which contact with the caregiver was common (e.g., home care)^60^ or in which continuity of care was offered (e.g., primary care) also facilitated providing caregiver support.^36^

### Caregiver Views

Seventeen studies reported on the caregiver views of the FROM programs reporting mostly positive feedback. Twelve of these collected qualitative data from interviews or focus groups,^25,27,30-32,35-37,48,51,54,56^ four were based on quantitative questionnaire feedback,^34,38,42,60^ and one used an open-ended questionnaire.^55^ FROM screening was generally welcomed,^25^ with caregivers reporting being grateful that someone was interested in their needs^37^ and that completing FROMs was worth their time and effort.^42,55^

#### Perceived Benefits

Peceived benefits mirrored those identified by clinicians.

##### Improved Communication

Caregivers reported that FROMs provided a starting point for expressing their needs.^25,27,51^

##### Focus on Caregivers’ Needs

FROMs provided space for caregivers to reflect on their situation and granted them permission to identify and consider their own needs.^27,30,48,54^ FROMs highlighted needs that caregivers may have been reluctant or found challenging to bring up.^25,30^

##### Validation of their Important Role

The FROM programs validated caregivers’ efforts and made them feel appreciated for their role.^25,27,30,48,54^ Some caregivers also noted that the screening was affirming, helping them recognize their own capacity to manage many of the challenges discussed.^48^

##### Linking to Resources

The FROM programs helped caregivers discover previously unknown services and support.^25,27,30,35,51,54^ Accessing these resources left caregivers feeling supported, reassured,^25,30-32,48,51,60^ secure, as though they had a security net,^32,51^ and less isolated.^25,31,48^ Some also became more proactive in addressing their needs,^31,35^ and others reported that care recipients may also feel reassured knowing their caregiver was being offered this support.^48^

#### Perceived Challenges

Caregivers reported that FROM screening might evoke strong emotions about their overwhelming journey,^27,35^ their future,^48,51^ or the death of the care recipient.^25,48^ Another challenge was balancing caregiver needs with those of the care recipient.^25,35,37,48^ Some caregivers hesitated to view themselves as legitimate participants^37^ or felt uncomfortable taking time away from care recipients during consultations.^35^ Some pointed out that the difference between their own needs (e.g., own health) and their needs related to being a ‘better caregiver' (e.g., providing personal care for the care recipient) could become blurred.^27^

#### Feedback on FROM Program Format and Delivery

In one study, caregivers noted that written formats might be overlooked amidst the abundance of paperwork they received.^36^ Using tablets for the screening^34,55,56^ and the telephone for the overall intervention^25^ received positive feedback. Ease of completion of FROMs was paramount,^25,27^ with caregivers appreciating self-completion for allowing more time to provide thoughtful responses.^25^ Tailoring FROMs to the caregiver’s specific situation, such as the stage of the care recipient’s illness, was also emphasized.^27,31,54^ Particular FROMs received positive feedback: the NAT-C was praised for its simplicity and clear rating system^35^; the distress screening tool (measuring distress, problems, and symptoms) provided a valuable outlet for caregivers to express emotions they rarely verbalize^32^; and the CSNAT was described as both simple and comprehensive.^25,27,30^

Caregivers stressed the importance of programs addressing both their needs and those of the care recipient.^56^ They valued having sufficient time to complete FROMs, preferably when it would not detract from caregiving responsibilities.^25^ While some caregivers preferred completing FROMs alone to avoid external influence,^27^ others appreciated having someone available to offer guidance, especially when unprepared for issues or emotions raised during the process.^51^ A well-structured and planned approach to screening and follow-up was generally well-received.^48^ Caregivers appreciated being the one to direct topics of discussion.^36,51^

In terms of who should respond to the FROM data, findings were mixed as to whether family physicians were well positioned to take on this role.^32,35,36^ In some studies, caregivers also discussed the idea of having a separate visit with the care recipients’ clinicians.^25,32,51,55^ They reported valuing the opportunity to speak privately and openly^32,51^ and wanting to complete the screening without having to share their concerns with the care recipient.^25^

Views on when to screen varied. Some reported wanting screening early on, whereas others felt that it was most important late in the caregiving trajectory when distress might be more elevated.^31,35^ Some suggested screening was most needed during transition points.^32^

### Efficacy of FROM Programs

#### Caregiver Distress and Strain

Six RCTs,^31-33,46,52,58^ one part-randomized stepped wedge cluster trial,^40^ and one quasi-experimental trial^29^ examined caregiver distress. Two^46,58^ reported a statistically significant reduction in the short-term (not sustained to the medium term). No significant reductions were reported in the three RCTs measuring distress in the long-term^31-33,52^ or the trial measuring distress 4-5 months post-bereavement.^40^

Of the three RCTs^46,58^ and one quasi-experimental study^29^ examining caregiver strain, one RCT^58^ found a significant reduction in caregiver strain in the medium-term. Across the studies, no positive effects were reported in the short- or long-term.

#### Caregiver Anxiety and Depression

Of the four RCTs^31,32,49,52^ measuring anxiety and six RCTs examining depression,^31-33,43,49,52^ none reported a significant reduction at any time point.

#### Caregiver Unmet Needs

Four RCTs^31,33,43,49^ examined unmet needs as an outcome and two found improvements.^33,43^ Of note, one of these FROM programs^33^ was intensive, offering 10-20 sessions with a clinical psychologist, including interventions tailored to the care recipient, caregiver, and/or dyad needs.

#### Preparedness for Caregiving

The two RCTs^32,58^ reporting on preparedness for caregiving found improvements at all data collection time points.

#### Caregiver QoL

Four RCTs^31,32,46,49^ and one quasi-experimental study^29^ examined caregiver QoL. Of the three reporting on overall QoL,^31,32,46^ none found significant improvements at any time point. No improvement was reported across the three studies^29,31,49^ measuring physical subscales of caregiver QoL/well-being. Five of the studies^29,31,32,46,49^ included emotional and/or psychological measures of QoL/wellbeing; of these, one RCT^31^ found a significant positive effect on emotional well-being.

## Discussion

The impact of caregiving is well-documented; the focus is now on establishing timely, tailored support to ensure caregivers have access to the assistance needed.^63^ This scoping review focused on the extent to which FROM programs among caregivers of care recipients with a chronic disease have been developed and evaluated. Findings suggest that: (a) two types of FROM programs have been commonly used: those focusing on unmet supportive care needs and others more aligned with traditional PROMs (e.g., DT); (b) the main goal of FROM screening has been to tailor interventions, not necessarily for symptom monitoring; (c) FROM screening appears acceptable, but efficacy is mixed at best; (d) caregivers focus on patients, highlighting the ongoing challenge of their engagement in interventions; (e) developing a stepped care approach to respond to FROMs might be more acceptable; (f) timing of the FROMs was not optimal; and (g) innovative models of FROM implementation have been used, including delivery by cancer helplines and through primary care. Each finding is now discussed in turn.

Two types of FROM programs were developed: (a) focused on unmet needs or (b) adapting traditional PROMs (DT, Hospital Anxiety and Depression scale, Edmonton Symptom Assessment System). Results do not suggest that one approach is superior to the other in terms of efficacy. A qualitative study identified five categories of FROs caregivers wish to address: emotional, physical symptoms, practical/social issues, cancer care, and financial concerns.^64^ The unmet needs approach addresses most of these areas, helping caregivers identify and prioritize their support needs. However, this approach is focused on tailoring interventions according to unmet needs, and not necessarily monitoring caregivers’ symptoms.

In contrast, programs adapting traditional PROMs primarily focused on emotional concerns or symptoms, which is aligned with other studies emphasizing that caregivers prioritize screening for these emotional outcomes.^52,63,64^ The DT, Hospital Anxiety and Depression scale, and Edmonton Symptom Assessment System Scale have been validated among caregivers.^32,41,42,59,65,66^ Co-collecting the same PROM and FROM data can provide a holistic understanding of the patient-caregiver dyads’ challenges, which in turn can further inform the tailoring of not only individual interventions, but also dyadic ones. Another potential advantage is that many of these PROMs are already used in clinical practice and their adaptation for caregivers could encourage broader implementation. Few FROM programs integrate both unmet needs and distress screening.^32,43,45,54,59,60^

Findings on FROM programs’ impact on caregivers’ health outcomes were inconsistent, despite their high acceptability. Low adherence may explain this; one study reported full adherence (≥80% completion) in only 18% of caregivers.^59^ Such adherence levels may influence proximal variables, like preparedness, but fail to affect harder-to-shift outcomes, such as anxiety. Proximal outcomes, which often operationalize the mechanism of action of FROM programs, did not consistently translate into changes in distal variables. Also, as most interventions had a broad focus (e.g., several domains of unmet needs), the typical health outcomes selected (e.g., anxiety, quality of life) might not capture the impact of such an all-encompassing approach.

It is well-known that screening for PROMs alone is not sufficient, and that follow-up care is essential to ensure efficacy.^67^ Across studies reviewed, even with dedicated clinicians available to address the FROM results, caregivers did not access this service as much as was anticipated. This is consistent with studies suggesting that less than half of caregivers with distress seek help or accept referrals to services.^43,59^ Lower than expected caregiver engagement emphasizes a need to “market” to caregivers the importance of meeting their needs and how this can contribute to caregivers maintaining their role. None of the reviewed studies reported introducing FROMs or had as part of their onboarding an explanation of how completing FROMs and accessing the services offered could ultimately improve care recipients’ outcomes. Incorporating an assessment of caregivers’ expectations—whether related to emotional support, practical assistance, respite care, or improved communication with clinicians —could further inform the design and delivery of FROM programs. By aligning program offerings with caregivers’ expectations, engagement may improve. In addition to promoting the importance of caregiver self-care, our team has found high caregiver acceptability for self-directed interventions (booklets and web-based intervention).^68-70^ Providing caregivers with these low intensity interventions, following FROMs, could increase their access to a (universal) minimal level of support. This suggestion is consistent with a previous study that found caregivers prefer to receive information from clinicians when their burden is high, but they prefer other information sources (e.g., internet) when burden is low.^71^ High-intensity support might then be offered within a stepped care approach,^72^ after caregivers have tried low-intensity interventions and only for those needing it. Stepped care might not only be more acceptable to caregivers, but also conserves scarce clinical resources.^73,74^

Another explanation for the lack of efficacy might be that the timing of the FROMs was not optimal. FROMs were most often administered once or twice for the primary purpose of tailoring follow-up interventions. This contrasts with the main purpose of PROMs, which is typically remote symptom monitoring, whereby longitudinal PROM data are used to determine symptom severity and the impact of intervention.^75^ The timing of FROMs was partially attributed to what was feasible to achieve given the length of the studies. In one study,^32^ FROMs took place every two months, and caregivers flagged that this did not correspond to key transition points for them, potentially underestimating caregivers’ distress.

Real-world implementation of PROMs has been the focus of much research for over two decades, and despite this, implementation and spread remain limited.^7,76-80^ FROMs implementation faces several unique barriers. For instance, many teams question whether it is within their scope of practice to respond to FROM data. Some of the studies reviewed used unique implementation models. For instance, Hawkes et al.^41^ explored the acceptability of using cancer telephone helplines to screen for PROMs and FROMs, based on who called. In another study,^43^ helpline oncology nurses called caregivers recruited from cancer center directly three times over a 4-month period. Needs identified by the DT were then addressed by the nurse who also facilitated navigation to other services. Mitchell et al.^49^ conducted an RCT of a family physician-based toolkit, which included a caregiver-reported needs checklist and a compendium of resources to help the family physician respond to caregivers’ needs. The intervention had mixed effects and seemed most efficacious among caregivers who were anxious or depressed at the outset of the study. The potentially limited efficacy of this FROM program may stem from caregivers’ preference to share their FROM data with the patient’s oncology team rather than their family physician. One primary reason for this preference is that oncology teams are perceived to have a deeper understanding of the specific challenges caregivers face in the context of cancer care, allowing for more tailored and relevant support.^64^

## Conclusion

### Future Research

Future research should explore the potential of dyadic PROM and FROM programs, as patients’ and caregivers’ interdependent symptoms, responses, and needs may amplify the impact of screening.^81^ More research is needed to delineate the mechanisms of action of FROM programs, determining whether targeting unmet needs and/or emotional FROs yields greater efficacy and corresponding distal outcomes that might be impacted. Using hybrid trials could also advance the implementation of FROM programs, documenting efficacious implementation strategies.^82^ Moreover, more evidence is needed to identify caregiver sub-groups that may benefit most from FROM programs, considering factors like age, care recipient’s illness, and context. For example, in the RCT conducted by Mitchell et al. (2013),^49^ while there were no between-group differences in the outcomes found from baseline to any time point, the findings indicated that certain groups with greater needs benefitted from the intervention. Caregivers who were clinically anxious reported significant improvement in mental health scores at 3-months and those who were clinically depressed avoided the significant worsening of anxiety that occurred in the control group at 6-months. Understanding these variations could help tailor programs for greater efficacy.

### Clinical Implications

Clinicians can advocate for integrating FROMs into routine care to identify caregivers needing support early. Potentially, they may rely on low-intensity intervention within a stepped care approach that can address caregivers’ unmet needs without adding strain to healthcare teams.

In conclusion, despite increased focus on family-centered care, the implementation of FROMs in cancer care remains scarce. This review provides concrete guidance to support the evidence-based development of FROM programs. Future research on the real-world implementation of FROM programs is needed.

## Data Availability

Data and materials used to conduct this study will be made available by emailing the corresponding author. There is no analytic code associated with this study.*

## Appendix

### Abbreviations

CSNAT-I: Carer Support Needs Assessment Tool intervention (CSNAT-I)
DT: Distress Thermometer
FROM: Family-reported Outcome Measure
NAT-C: Needs Assessment Tool for Caregiver
PRISMA-ScR: Preferred Reporting Items for Systematic Reviews and Meta-Analyses—Scoping Review Extension
PRO: Patient-reported Outcome
PROM: Patient-reported Outcome Measure
QoL: Quality of Life, RCT: Randomized Controlled Trial
